# Comparative transcriptome analysis reveals different defence responses during the early stage of wounding stress in *Chi-Nan* germplasm and ordinary *Aquilaria sinensis*

**DOI:** 10.1186/s12870-022-03821-4

**Published:** 2022-09-29

**Authors:** Feifei Lv, Yun Yang, Peiwen Sun, Yan Zhang, Peiwei Liu, Xiaohong Fan, Yanhong Xu, Jianhe Wei

**Affiliations:** 1 Hainan Provincial Key Laboratory of Resources Conservation and Development of Southern Medicine & Key Laboratory of State Administration of Traditional Chinese Medicine for Agarwood Sustainable Utilization, Hainan Branch of the Institute of Medicinal Plant Development, Chinese Academy of Medical Sciences and Peking Union Medical College, Haikou, 570311 China; 2 Key Laboratory of Bioactive Substances and Resources Utilization of Chinese Herbal Medicine, Ministry of Education & National Engineering Laboratory for Breeding of Endangered Medicinal Materials, Institute of Medicinal Plant Development, Chinese Academy of Medical Sciences and Peking Union Medical College, Beijing, 100193 China

**Keywords:** *Aquilaria sinensis*, Transcriptome, Defence response, Differentially expressed genes, The agarwood-producting capacity

## Abstract

**Background:**

Agarwood is a valuable Chinese medicinal herb and spice that is produced from wounded *Aquilaria* spp., is widely used in Southeast Asia and is highly traded on the market. The lack of highly responsive *Aquilaria* lines has seriously restricted agarwood yield and the development of its industry. In this article, a comparative transcriptome analysis was carried out between ordinary *A. sinensis* and *Chi-Nan* germplasm, which is a kind of *A. sinensis* tree with high agarwood-producing capacity in response to wounding stress, to elucidate the molecular mechanism underlying wounding stress in different *A. sinensis* germplasm resources and to help identify and breed high agarwood-producing strains.

**Results:**

A total of 2427 and 1153 differentially expressed genes (DEGs) were detected in wounded ordinary *A. sinensis* and *Chi-Nan* germplasm compared with the control groups, respectively. KEGG enrichment analysis revealed that genes participating in starch metabolism, secondary metabolism and plant hormone signal transduction might play major roles in the early regulation of wound stress. 86 DEGs related to oxygen metabolism, JA pathway and sesquiterpene biosynthesis were identified. The majority of the expression of these genes was differentially induced between two germplasm resources under wounding stress. 13 candidate genes related to defence and sesquiterpene biosynthesis were obtained by WGCNA. Furthermore, the expression pattern of genes were verified by qRT-PCR. The candidate genes expression levels were higher in *Chi-Nan* germplasm than that in ordinary *A. sinensis* during early stage of wounding stress, which may play important roles in regulating high agarwood-producing capacity in *Chi-Nan* germplasm.

**Conclusions:**

Compared with *A. sinensis*, *Chi-Nan* germplasm invoked different biological processes in response to wounding stress. The genes related to defence signals and sesquiterepene biosynthesis pathway were induced to expression differentially between two germplasm resources. A total of 13 candidate genes were identified, which may correlate with high agarwood-producting capacity in *Chi-Nan* germplasm during the early stage of wounding stress. These genes will contribute to the development of functional molecular markers and the rapid breeding highly of responsive *Aquilaria* lines.

**Supplementary Information:**

The online version contains supplementary material available at 10.1186/s12870-022-03821-4.

## Background

Agarwood is a precious Chinese traditional medicine with obvious medicinal uses as a sedative and carminative and in the relief of gastric problems [1]. As a rare spice, agarwood also plays a role in fragrances, incense, aromatherapy and religious ceremonies [2, 3]. Agarwood is the resinous heartwood derived from wounded *Aquilaria* spp. trees, which is an angiosperm in the *Thymelaeaceae* family [4]. There are more than 20 species of *Aquilaria* spp. to produce agarwood in the world, among which *A. sinensis* is the only legal resource for agarwood production in China [3, 5]. For thousands of years, a large number of wild *Aquilaria* spp. plants have been cut down to increase profits, which has led to resource depletion [6]. Thus, wild *Aquilaria* spp. resources are scarce, and the species have been listed on the IUCN red list as endangered species [7]. To meet market demand and conserve agarwood resources, *Aquilaria* spp. plants have been widely cultivated to allow sustainable agarwood production [8]. However, it is difficult for *Aquilaria* spp. planted worldwide to produce agarwood, and the efficiency of agarwood production has been very low in recent decades. Hundreds of millions of *Aquilaria* spp. worldwide do not produce agarwood efficiently and quickly, which seriously restricts the use and industrial development of agarwood. Although our laboratory has developed a whole-tree agarwood-inducing technique that is widely used worldwide, the production of agarwood is less than 15% of the weight of stems or trunks in each tree, and the content of alcohol extract after 1–2 years of agarwood production is less than 25% on average [9]. In addition, fungal infection and cutting and drilling techniques are also applied to produce agarwood [10, 11]. During the popularization and application of the artificial injury techniques, the *Chi-Nan* germplasm was found. By DNA barcoding, the *Chi-Nan* germplasm was identified as *Aquilaria sinensis (Lour.) Spreng*, and is considered a new *A. sinensis* germplasm, that can produce agarwood by cold drilling which is the simplest technique. The production and alcohol extraction of agarwood can reach more than 30 and 35%, respectively. However, the chemical composition of agarwood produced from the *Chi-Nan* germplasm was quite different from that of ordinary *A. sinensis* [12]. Therefore, breeding and proper utilization of *Chi-Nan* germplasm resource will improve the yield and quality of agarwood on the market.

Sesquiterpene is one of the main compounds in agarwood [13, 14] and plays vital roles in plant defence against external stimuli [15, 16]. Sesquiterpene is not found in healthy *A. sinensis* and can only be formed in stems, branches or roots subjected to wounding stress. The synthesis of a variety of sesquiterpenoids is induced in wounded *A. sinensis* [14]. The high content of sesquiterpene and the diversity of their components determine the resin yield and unique fragrance of agarwood to some extent. Sesquiterpene has been an important criterion to evaluate agarwood quality [14]. The synthetic efficiency of sesquiterpenoids induced by injury could reflect the capacity of agarwood production in *A. sinensis*. Similar to other plants, sesquiterpene biosynthesis is mainly based on the MVA pathway in the cytoplasm and the MEP pathway in plastids in wounded *A. sinensis* [17, 18]. By transcriptome sequencing, 30 putatively encoded enzymes have been predicted to be involved in sesquiterpene biosynthesis, including sesquiterpene synthases and HMG-CoA reductase [19, 20]. Some enzymes have been cloned, and their functions have been verified [19, 21–23]. In the sesquiterpene biosynthesis pathway, sesquiterpene synthase is the rate-limiting enzyme that can catalyse different rearrangements of carbocation intermediates in substrates, such as FPP, to form diverse sesquiterpenoids [24–26]. The activity of sesquiterpene synthase is closely related to gene expression. Previous studies have confirmed that external stimulation triggers an H_2_O_2_ burst, activates JA signalling, delivers wounding signals to transcription factors (WRKY\MYC2), and further induces sesquiterpene synthase gene expression [27–30]. In addition, cytochrome P450 enzymes play important roles in modifying sesquiterpene scaffolds [31]. Overall, wound-inducible sesquiterpene biosynthesis requires defence signals and multiple synthases for collaborative regulation.

In practice, the efficiency and quality of agarwood synthesis by wound induction in *Chi-Nan* germplasm is higher than that in ordinary *A. sinensis* [12]. Thus, *Chi-Nan* germplasm is a highly responsive *Aquilaria* line. To explore the mechanism of high agarwood-producing capacity in *Chi-Nan* germplasm and compare the difference in agarwood formation between ordinary and highly responsive germplasm in *A. sinensis*, we investigated the early defence response by transcriptome sequencing. Comparative dynamic analysis of the number of DEGs and functional enrichment suggested that the differences between the ordinary *A. sinensis* and *Chi-Nan* germplasms were present in the early stage after wounding stress. JA signal-, oxygen metabolism- and sesquiterpene biosynthesis-related DEGs were identified. A total of 13 candidate genes were selected, which had significantly different expression patterns between the two germplasm resources, suggesting that the may be vital to the regulation of agarwood-producing capacity in *A. sinensis*. These findings are useful for further characterization of these candidate genes and helpful for quickly identifying and breeding excellent agarwood-producing germplasm of *A. sinensis*.

## Results

### Induced-biosynthesis of secondary metabolites in different germplasm resources after injury

**Fig. 1:**
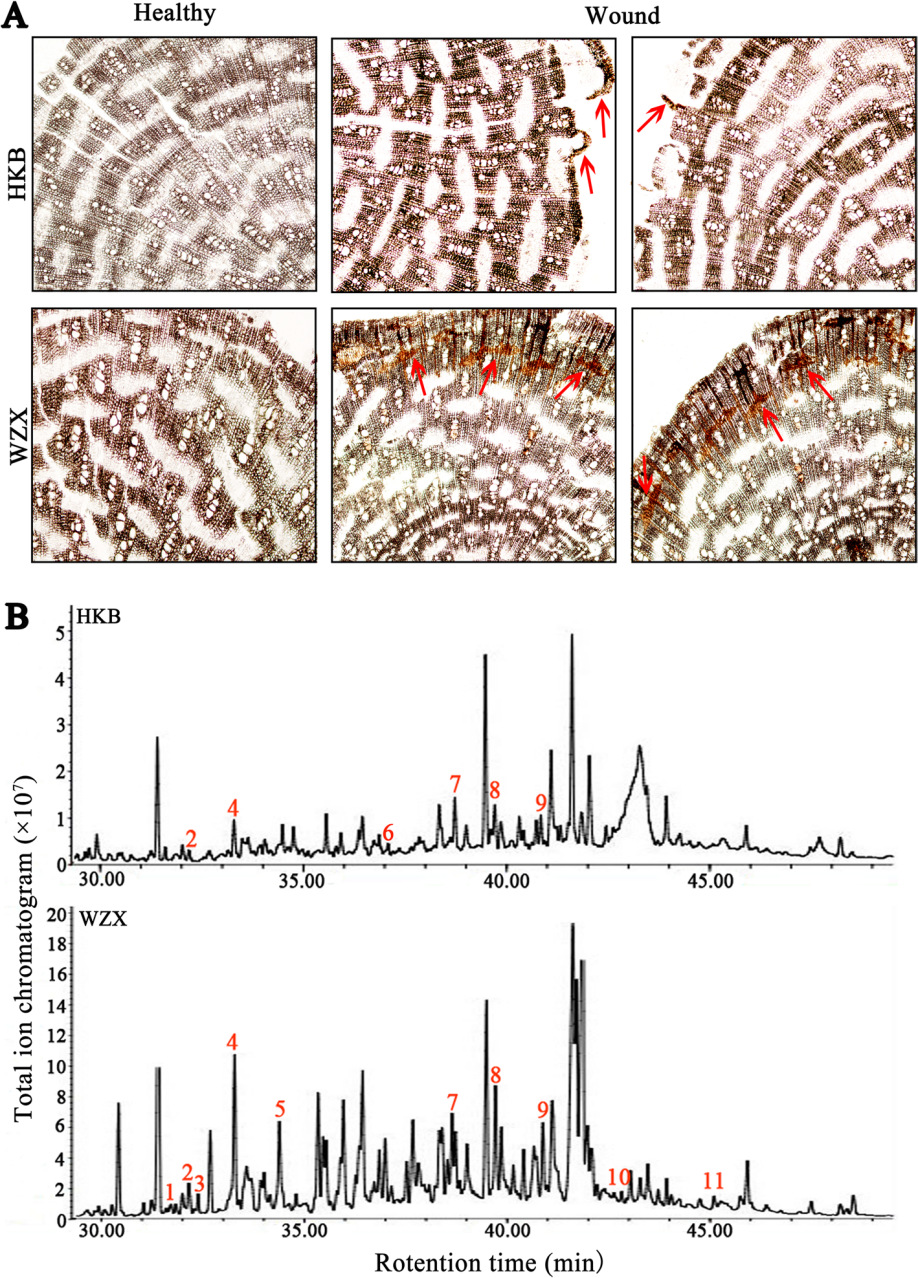
Comparative analysis of the formation of secondary metabolites after wounding stress in ordinary *A. sinensis* and *Chi-Nan* germplasm (**A**) Microstructure (10×) difference between branches in ordinary *A. sinensis* and *Chi-Nan* germplasm. The red arrow points to the secondary metabolites produced after the injury. **B** Mass spectra of sesquiterpene compounds in two germplasm resources after the injury. The peaks marked with a numerical value are the sesquiterpenes compounds. Chromatographic peaks represent compounds as follows 1 beta.-guaiene, 2 beta.-humulene; 3 delta.-selinene; 4 gama.-eudesmol; 5 caryophyllene oxide; 6 aromandendrene; 7 alpha.-Farnesene; 8 longifolenaldehyde; 9 isoaromadendrene; 10 caryophyllene-(I1); 11 alloaromadendrene. HKB represents the ordinary *A. sinensis*, WZX represents *Chi-Nan* germplasm

Agarwood is a kind of resinous wood formed after damage to *A. sinensis*. As a secondary metabolite, agarwood can be produced and can accumulate in the interxylophem of stems, branches or roots after wounding stress [32]. In this study, the microscopic structure of branches before and after injury was observed in ordinary *A. sinensis* and *Chi-Nan* germplasm. As shown in Fig. 1A, yellowish-brown resin was observed in the phloem of wounded ordinary *A. sinensis* and *Chi-Nan* germplasm; this resin was not found in any healthy branches. Moreover, the yellowish-brown substance accumulated more in wounded *Chi-Nan* germplasm than in wounded ordinary *A. sinensis*. These results suggested that the yellowish-brown resin could form after injury to both ordinary *A. sinensis* and *Chi-Nan* germplasm, but the efficiency and yield of resin synthesis differed significantly between the two germplasm resources.

Furthermore, GC–MS detected sesquiterpenoids and volatile substances from wounded branches of both germplasms resources. It was revealed that the wounded branches contained various sesquiterpenes and derivatives in both germplasms (Fig. 1B), whereas these were not detected in any healthy branches (no data). In the chromatogram, each peak represents a compound that has been detected. In wounded ordinary *A. sinensis*, we detected six sesquiterpene compounds: β-humulene, γ-eudesmol, aromandendrene, α-farnesene, longifolenaldehyde, and isoaromadendrene, respectively. In wounded *Chi-Nan* germplasm, in addition to the sesquiterpenoids detected in ordinary *A. sinensis*, we also detected β-guaiene, δ-selinene, caryophyllene oxide, caryophyllene-(I1) and alloaromadendrene (Fig. 1B), suggesting that *Chi-Nan* germplasm can produce more abundant sesquiterpenoids under wounding stress. Furthermore, we compared the relative amounts of sesquiterpenoids detected and found that the amount of sesquiterpenoids in *Chi-Nan* germplasm was higher than that in ordinary *A. sinensis* (Table S2). Overall, the results indicated that both germplasms could produce agarwood resin after wounding stress, but the efficiency and yield of agarwood formation induced in *Chi-Nan* germplasm were obviously higher than those in ordinary *A. sinensis*, that is, the defence response mechanism after injury was significantly different between the two germplasm resources.

### RNA-sequencing analysis and global comparison of transcriptomes

**Fig. 2:**
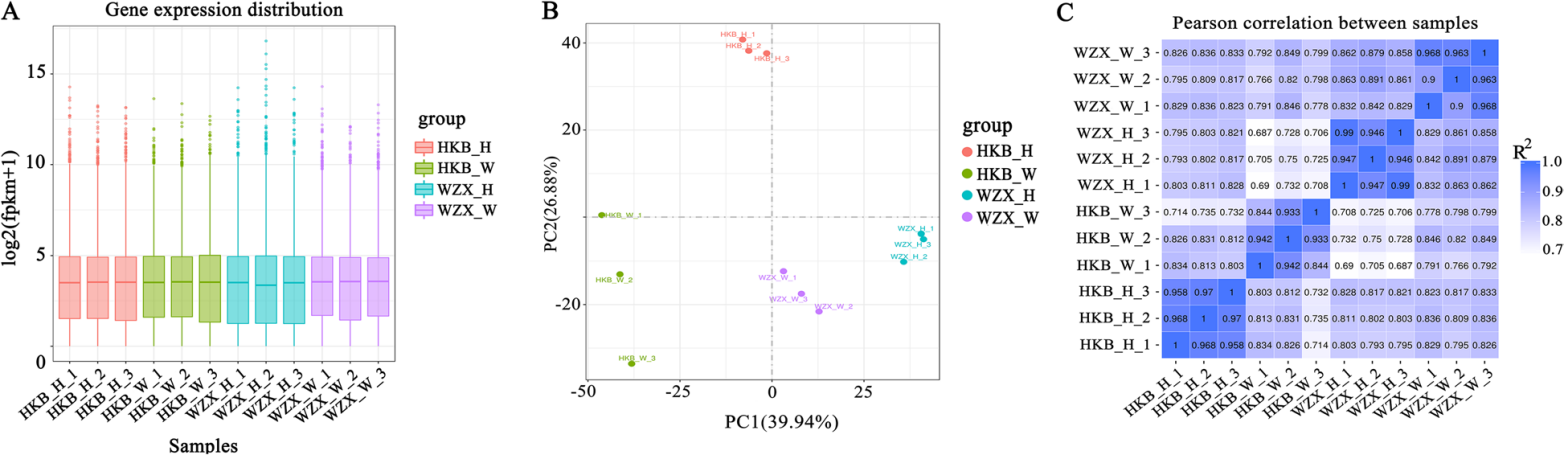
Overview of the Illumina transcriptome sequencing. **A** Gene expression levels of all the 12 libraries. **B** Principal component analysis (PCA) of the 12 samples based on gene expression levels. **C** Heatmap clustering showing the sample correlation analysis of the 12 sequenced samples. HKB represents the ordinary *A. sinensis*, WZX represents *Chi-Nan* germplasm, H represents Healthy, W represents Wounded

To develop a method for rapid breeding, we explored the molecular basis of the difference in the early defence response induced by wounding stress based on different agarwood biosynthesis levels. RNA-seq analysis was conducted to generate transcriptome profiles. Twelve libraries were constructed and analysed. A total of 77.36 Gb of clean data were obtained, and an average of 6.44 Gb of high-quality clean reads were obtained for each sample after removing the low-quality reads. The GC content of the sequence data was approximately 46.55%, and the Q30 values were all above 93.66%, indicating that the quality and accuracy of the sequencing data were sufficient for further analyses. A total of 91.73–95.94% clean reads were mapped to the *A. sinensis* reference genome (BioProject ID: PRJNA524272), among which 82.5–90.24% were uniquely matched (Table S3). The range of the fragments per kilobase of exon per million fragments mapped (FPKM) values in all 12 libraries was examined with box plots (Fig. 2A). Principal component analysis (PCA) and Pearson correlation coefficients (R^2^ > 0.84) of three biological replicates for each treatment for both the ordinary *A. sinensis* and *Chi-Nan* germplasm were high (Fig. 2B-C), suggesting that the RNA-seq data were of high quality and consistency.

### Screening of differentially expressed genes

**Fig. 3:**
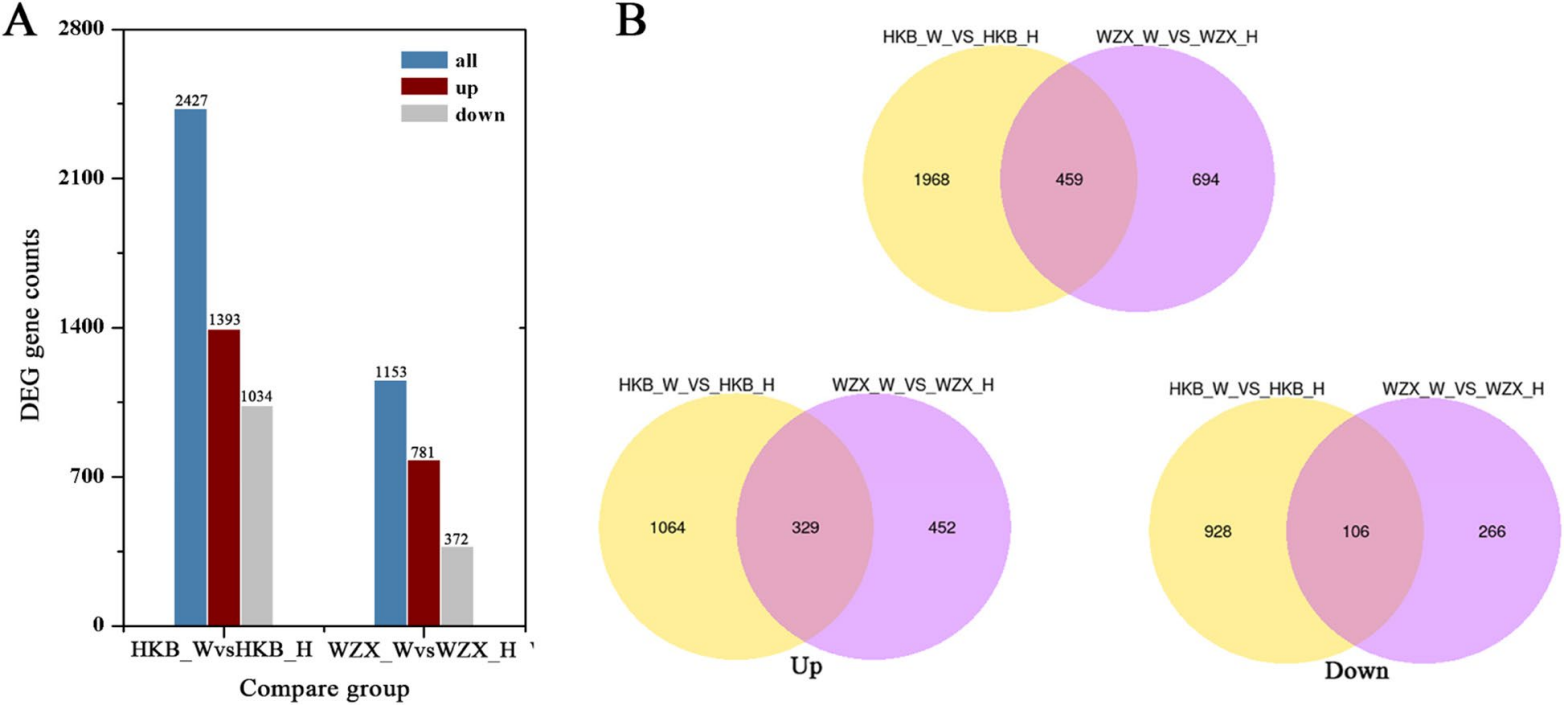
Statistical analyses of DEGs in ordinary *A. sinensis* and *Chi-Nan* germplasm*.* **A** The number of DEGs in different comparisons**. B** Venn diagrams of the overlapped DEGs identified in ordinary *A. sinensis* and *Chi-Nan* germplasm under wounding stress including total overlapped DEGs, the up overlapped DEGs and the down overlapped DEGs in two comparisons. HKB represents the ordinary *A. sinensis*, WZX represents *Chi-Nan* germplasm, H represents Healthy, W represents Wounded

The DEGseq method was used to analyse the gene expression patterns between ordinary *A. sinensis* and *Chi-Nan* germplasm at 0 and 6 h after wounding stress. During the early stage of wound-induced agarwood production, the expression levels of genes related to sesquiterpene synthesis were strongly induced at 6 h. DEGs were detected in this study with a *P* value ≤0.05 and |log2 (fold change)| > 2. In total, 2427 and 1153 differentially expressed genes were identified in ordinary *A. sinensis* and *Chi-Nan* germplasm, respectively. Among the DEGs in ordinary *A. sinensis*, 1393 were upregulated, and 1034 were downregulated. Moreover, there were 781 upregulated genes and 372 downregulated genes in the *Chi-Nan* germplasm comparison. (Fig. 3A).Venn diagram analysis showed that 459 DEGs overlapped in the ordinary *A. sinensis* and *Chi-Nan* germplasm comparisons, among which 329 common genes were upregulated and 106 common genes were downregulated (Fig. 3B).

### Functional enrichment analyses of DEGs in ordinary *A. sinensis* and *chi-nan* germplasm resources

**Fig. 4:**
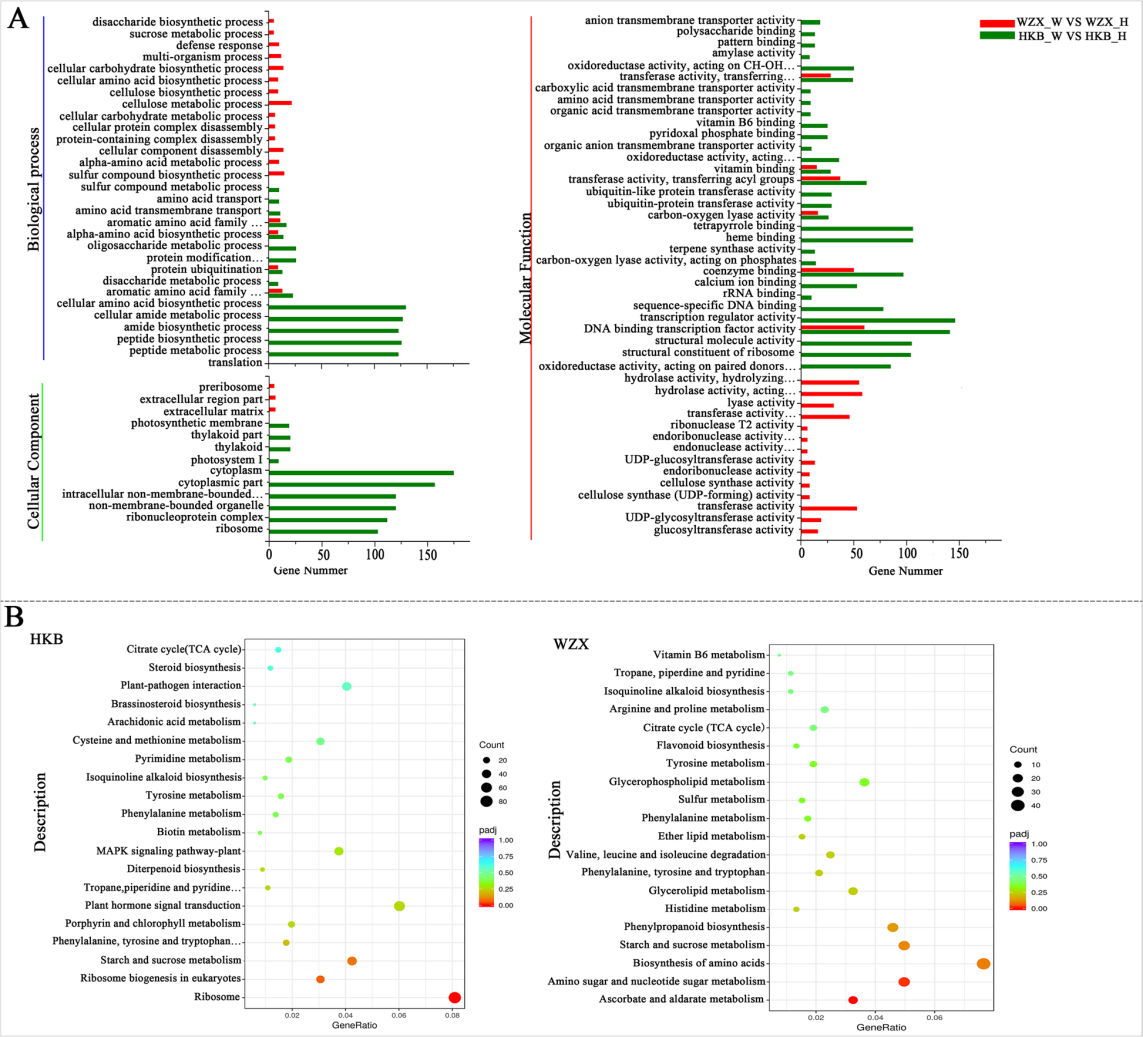
GO terms and KEGG pathways enriched in ordinary *A. sinensis* and *Chi-Nan* germplasm. **A** the GO terms enriched in DEGs of ordinary *A. sinensis* and *Chi-Nan germplasm* before and after wounding stress. **B** Enriched KEGG pathways of DEGs in two germplasm resources. HKB represents the ordinary *A. sinensis*, WZX represents *Chi-Nan* germplasm

To deeply understand the difference in biological mechanisms during the early stage of wounding, GO enrichment analyses of the DEGs in ordinary *A. sinensis* and *Chi-Nan* germplasm were conducted, respectively. As shown in Fig. 4A, the DEGs in ordinary *A. sinensis* were assigned to 56 terms belonging to three categories: biological process (15 terms), cell components (10 terms), and molecular function (31 terms) (*P ≤* 0.05). 41 GO terms were identified in *Chi-Nan* germplasm (*P ≤* 0.05), including 18 biological process terms, 3 cell component terms and 20 molecular function terms. In terms of the numbers of DEGs related to GO terms, the ordinary *A. sinensis* induced more genes related to more GO terms than the *Chi-Nan* germplasm. It was notable that GO terms enriched in the three categories were quite different in two the comparisons, for example, ordinary *A. sinensis* germplasms had “oxidoreductase activity”, “terpene synthase” and “transmembrane transporter activity” in molecular function while the *Chi-Nan* germplasm was not enriched in these terms. However, “glucosytransferase activity” and “hydrolase activity” enriched in molecular processes and “defence response” enriched in biological processes in the *Chi-Nan* germplasm also did not appear in ordinary *A. sinensis*. These results indicated that the wound response pathway and pattern of *Chi-Nan* germplasm are significantly different from those of ordinary *A. sinensis* during the early stage of wounding stress. In this process, ordinary *A. sinensis* could induce more genes related to physiological processes to resist injury.

KEGG analysis was performed to identify the potential biological pathways of genes represented in the transcriptome during the early stage of wounding stress in *A. sinensis*. A total of 111 and 109 pathways were enriched for DEGs in ordinary *A. sinensis* and *Chi-Nan* germplasm, respectively (Table S4). The DEGs of the two comparisons were almost enriched in 109 KEGG pathways, except for the “glycosylphosphatidyl inositol (GPI)-anchor biosynthesis” and “brassinosteroid biosynthesis” pathways, but the number of DEGs in each pathway was different between the ordinary *A. sinensis* and *Chi-Nan* germplasm (Table S4). The top 20 pathways in the two comparisons are shown in Fig. 4B, only the “Ribosome” pathway in ordinary the *A. sinensis* comparison and the “ascorbate and aldarate metabolism” “amino sugar and nucleotide sugar metabolism” pathways in the *Chi-Nan* germplasm comparison were significantly enriched with a cut-off value of padj< 0.05. The primary metabolism pathway including “starch and sucrose metabolism” and “citrate cycle (TCA cycle)” , and secondary metabolism pathway including “phenylalanine metabolism”, “tyrosine metabolism”, and “isoquinoline alkaloid biosynthesis” were enriched in top the 20 pathways in both two germplasms resources and might play major roles to involve in the early regulation of wounding stress in *A. sinensis*. Additionally, as an important defence signal, JA signal-related genes belonging to the “alpha-linolenic acid metabolism” and “plant hormone signal transduction” pathways were also enriched in the two comparisons. “Terpenoid backbone biosynthesis” pathways containing genes for sesquiterpene backbone biosynthesis were also enriched (Table S3), suggesting that ordinary *A. sinensis* and *Chi-Nan* germplasm can both induce JA defence signaling and terpenoid biosynthesis genes to resist wounding stress during the early stage of injury.

### Gene expression maps analysis in sesquiterpene biosynthesis and JA pathway

**Fig. 5:**
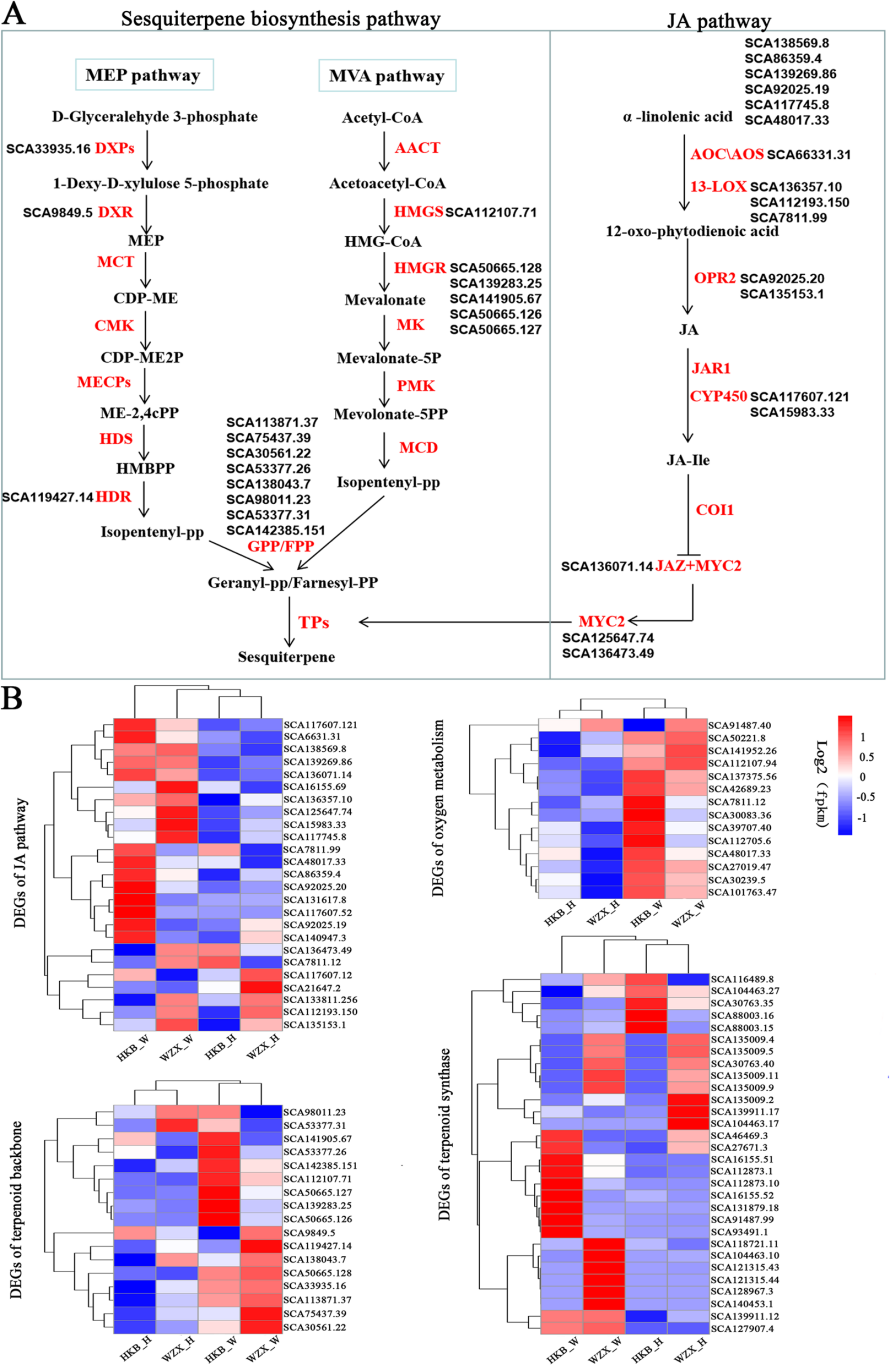
Heat maps of the genes involved in the sesquiterpene biosynthesis, JA pathway and oxygen metabolism in two germplasm resources. **A** Sesquiterpene biosynthesis and JA pathways. The enzymes were involved in the biosynthesis of sesquiterpene including acetoacetyl-CoA thiolase (AACT), hydroxymethylglutaryl-CoA synthase (HMGS), hydroxymethylglutaryl-CoA reductase (HMGR), mevalonate kinase (MK), phosphomevalonate kinase (PMK), mevalonate-diphosphate decarboxylase (MCD), farnesyl pyrophosphate synthase (FPPS), Geranylgeranyl pyrophosphate synthase (GPP), terpene synthase (TPs). 1-deoxy-D-xylulose-5-phosphate synthase (DXS), 1-deoxy-D-xylulose-phosphate reductoisomerase (DXR), 4-diphosphocytidyl-2C-methyl-D-erythritol kinase (CMK), 2C-methyl-D-erythritol 2,4-diphosphate synthase (MECS), 1-hydroxy-2-methyl-butenyl 4-diphosphate synthase (HDS), 1-hydroxy-2-methyl-butenyl 4-diphosphate reductase (HDR). The genes were involved in JA pathway including allene oxide cyclase (AOC), allene oxide synthase (AOS), 13-lipoxygenase (13-LOX), OPDA reductase (OPR2), jasmonate resistant synthase (JAR), cytochrome p450 (CYP450), coronatine insensitive (COI1), jasmonate ZIM-domain protein (JAZ), basic helix–loop–helix transcription Factor (MYC2). **B** The hierarchical clustering was carried out to Heat-map cluster analysis on the FPKM value of genes. Heat map shows values (− 1.5 to 1.5) about homogenization processing (Z-score) on rows ranging from blue (low expression) to red (high expression). HKB represents the ordinary *A. sinensis*, WZX represents *Chi-Nan* germplasm. H represents Healthy, W represents Wounded

Sesquiterpene is one of the main components of agarwood, which is the resin wood produced by wounding stress in *A. sinensis* [12] sesquiterpene biosynthesis was different between wounded ordinary *A. sinensis* and *Chi-Nan* germplasm (Fig. 1B). To analyze the difference in wound-induced sesquiterpene biosynthesis between *Chi-Nan* germplasm and ordinary *A. sinensis*, we focused on DEGs enriched in sesquiterpene biosynthesis pathway. Similar to other species, sesquiterpene biosynthesis was based on MEP and MVA pathway in *A. sinensis* (Fig. 5A). Seventeen terpenoid backbone and 30 terpene synthase genes were identified in transcriptome. Except *SCA98011.23* and* SCA53377.31*, terpenoid backbone genes expression were up-regulated after wounding stress, but the significantly expressed genes were different between two germplasm resources. The expression levels of *SCA141905.67*, *SCA53377.36*, *SCA142385.151*, *SCA112107.71*, *SCA50665.127*, *SCA13928335* and *SCA50665.126* involved in MEP pathway were significantly increased in wound ordinary *A. sinensis,* but not in *Chi-Nan* germplasm. Compared with ordinary *A. sinensis*, *SCA9849.5*, *SCA119427.14*, *SCA138043.7*, *SCA50665.128*, *SCA33935.16*, *SCA113871.37*, *SCA75437.39* and *SCA30561.22* participating in MEP pathway were induced to higher express*.* Meanwhile, we further found the induced expression levels of terpene synthase genes were also different between two germplasm resources, for example, *SCA112873.10*, *SCA161155.52*, *SCA131879.18*, *SCA91487.99*, *SCA27671.3* and *SCA93491.1* were induced to express in ordinary *A. sinensis*, and *SCA118721.11*, *SCA104463.10*, *SCA121315.43*, *SCA121315.44*, *SCA128967.3* and *SCA140453.1* were up-regulated in *Chi-Nan* germplasm (Fig. 5B). These differentially induced expression of “terpenoid backbone biosynthesis” and “terpene synthase” genes may be the direct factors for the difference in wound-induced biosynthesis of sesquiterpene between the two germplasms resources.

Previous studies showed that JA signal was vital signal to respond to wounding stress and could regulate sesquiterpene synthase gene *ASS1* expression during wound-induced agarwood formation in *A. sinensis* [19]. In this study, 25 DEGs related to JA pathway were identified, among which 10 genes were induced to expression in both ordinary *A. sinensis* and *Chi-Nan* germplasm under wounding stress, 8 genes expression levels were up-regulated only in ordinary *A. sinensis,* 4 genes showed opposite expression patterns in the two comparisons, other 3 genes have not any change after wounding stress (Fig. 5B). It was confirmed that the response mode of JA signal was different between two germplasm resources to resist injury and promote sesquiterpene biosynthesis during early stage of wounding stress. It’s worth noting that *AsMYC2* (*SCA125647.74* and *SCA136473.49*) that is the transcription factors in JA pathway for promoting *ASS1* expression [27] were noticeably elevated in *Chi-Nan* germplasm*,* which may correlated with high content of sesquiterpene in *Chi-Nan* germplasm.

In addition, oxygen metabolism that is an important signal to activate JA in the process of wound-induced agarwood formation in *A. sinensis* [29, 33] was enriched. 14 oxygen metabolism genes were also identified and the gene expression levels were up-regulated in both wound ordinary *A. sinensis* and *Chi-Nan* germplasm. Only 4 genes were induced to higher express in *Chi-Nan* germplasm comparison than that in ordinary *A. sinensis* (Fig. 5B). It was further indicated that the wound response was weaker and the physiological metabolic activity was less in *Chi-Nan* germplasm, compared with ordinary *A. sinensis*.

### Construction of gene co-expression networks in two *A. sinensis* germplasms during early stage of wounding stress

**Fig. 6:**
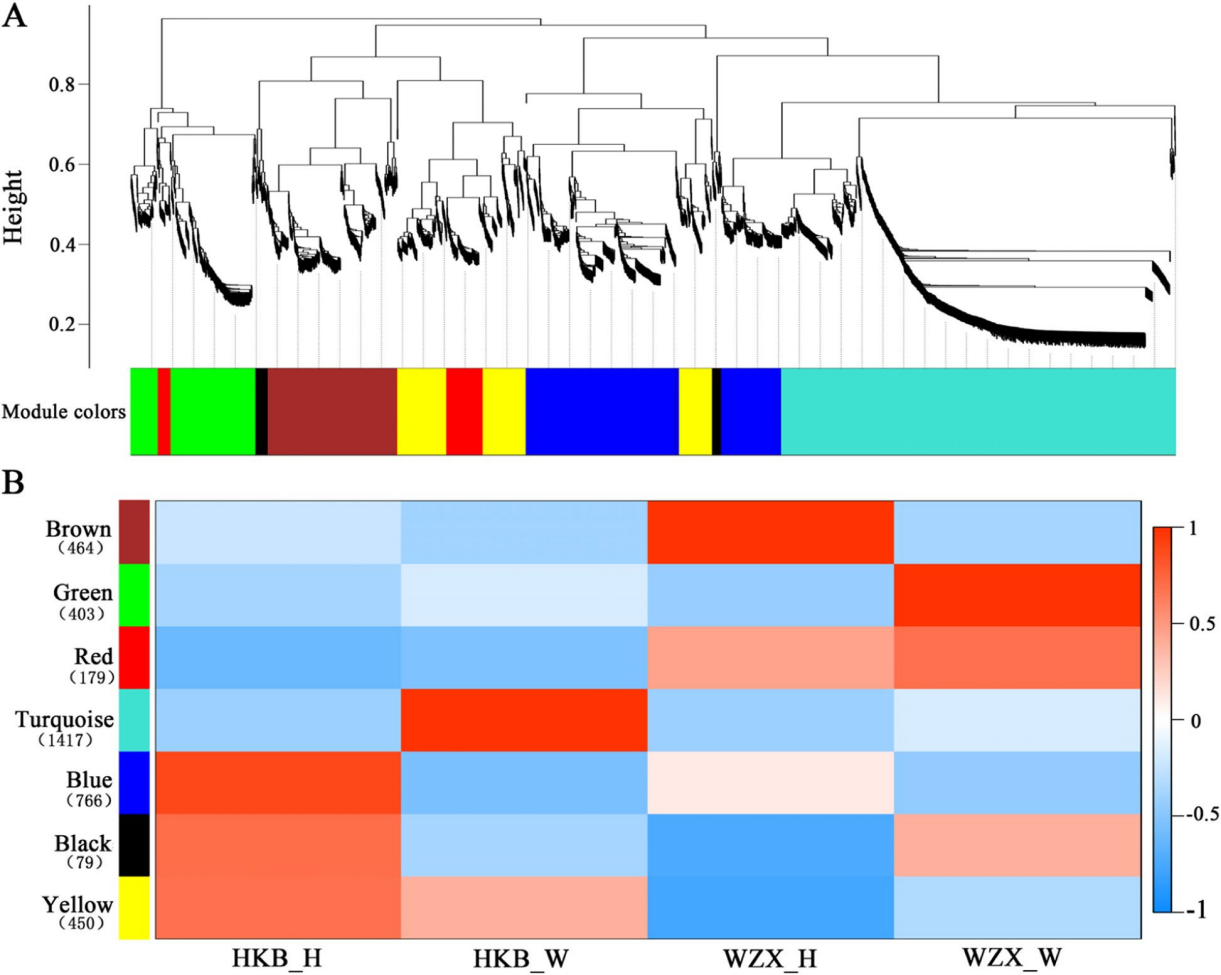
Weighted gene co-expression network analysis of relative DEGs. **A** Hierarchical cluster dendrogram showed co-expression modules. Each leaf (short vertical line) in the tree represented one gene. The genes were clustered based on dissimilarity measure. The major tree branches, corresponded with the color rows below the dendrogram, constituted the modules. **B** Module-sample association analysis. Each row corresponded to a module, and each column corresponded to a sample. The number of gene in each module was displayed in parentheses on the left of each row. HKB represents the ordinary *A. sinensis*, WZX represents *Chi-Nan* germplasm. H represents Healthy, W represents Wounded

To obtain an insight for the molecular mechanisms difference between two *A. sinensis* germplasm resources during early stage of wounding stress, WGCNA analysis was carried out to construct the gene co-expression network. All 3771 DEGs were assigned into 7 distinct modules labeled with different colors (Fig. 6A), except 13 of which cannot be assigned into any module were discarded. The highly interconnected genes with similar expression changes were clustered into a module. The correlation between modules and samples was analyzed, the green and turquoise modules were showed highly correlation with wounded *Chi-Nan* germplasm and oridnary *A. sinensis,* respectively (Fig. 6B). Noticeably, the red and yellow module both performed opposite expression patterns between two germplasms resources, but genes expression levels in *Chi-Nan* germplasm were higher than that in ordinary *A. sinensis* in red module (Fig. 6B), indicating these genes were related to high production in *Chi-Nan* germplasm.

Furthermore, we performed KEGG enrichment analysis of green and turquoise modules to explore physiological metabolic difference between wounded two germplasm resources. KEGG pathways enriched in green module were all enriched in turquoise module (Table S5). In addition, some secondary metabolism and physiological activity pathway containing “isoquinoline alkaloid biosynthesis” “phenylalanine metabolism” and “photosynthesis” were enriched in turquoise module (Table S5), which were accordance with the KEGG analysis of DEGs in two germplsms resources. It is further confirmed ordinary *A. sinensis* correlated more physiological activities to resist wounding stress. Meanwhile, “alpha-linolenic acid metabolism” involved in JA biosynthesis and “terpenoid backbone biosynthesis” and “terpene synthase” involved in sesquiterpene biosynthesis were all enriched in green and turquoise modules (Table S5). In “alpha-linolenic acid metabolism” pathway, *SCA15983.33* (*P450*) and *SCA 136357.10* (*Lox*) showed highly correlation with wound *Chi-Nan* germplasm*,* suggesting *SCA15983.33* and *SCA 136357.10* are significant genes to regulate defense response in *Chi-Nan* germplasms. “terpenoid backbone biosynthesis” genes including *SCA113871.37* (*Geranylgeranyl pyrophosphate synthase*), *SCA50665.128* (*Hydroxymethylglutaryl-coenzyme A reductase*) and *SCA18745.55* (*Mevalonate 5-diphosphate decarboxylase*) (Table S5) and some terpene synthase genes (*SCA127907.4*, *SCA121315.43*, *SCA128967.3*, *SCA104463.10*) were also enriched in green module, which highly correlated with wounded *Chi-Nan* germplasm. In addition, some terpene synthase genes (*SCA135009.11.add*, *SCA 135009.4*, *SCA135009.5*, *SCA30763.4* were clustered in red module) were higher expression levels in *Chi-Nan* germplasm than that in ordinary *A. sinensis*. In total, there were 13 genes were highlighted after WGCNA and interaction network analyses, which are considered to be candidate genes for diversity and high production of sesquiterpene biosynthesis in *Chi-Nan* germplasm resources*.*

### Gene expression validation by qRT-PCR

**Fig. 7:**
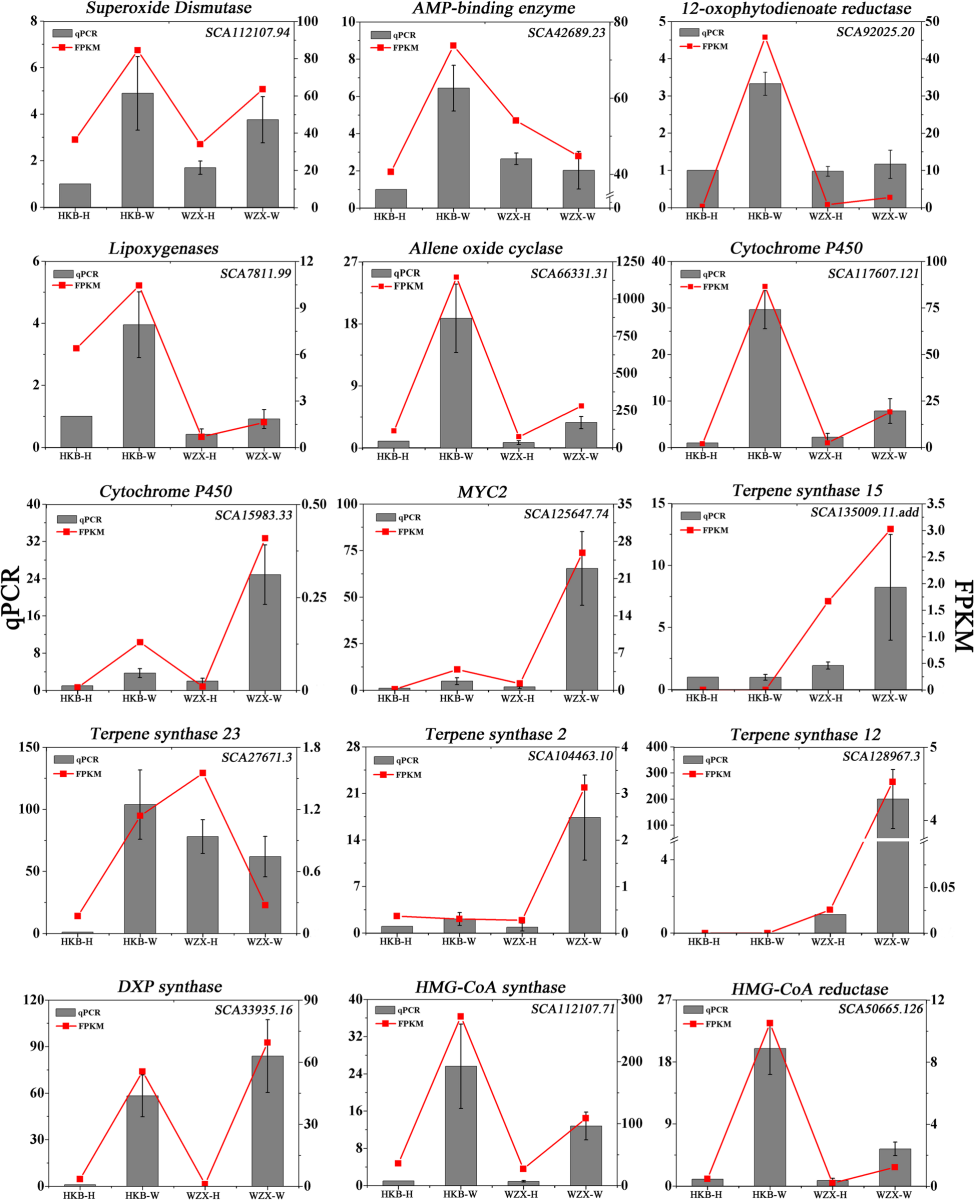
The expression of genes related to sesquiterpiodbiosynthesis were validated by qRT-PCR. Gray columns show the qPCR results of 15 unigenes involved in defense response and sesquiterpene biosynthesis, represent the mean ± SD of three biological replicates. The red lines show the FPKM values of these unigenes. HKB represents the ordinary *A. sinensis*, WZX represents *Chi-Nan* germplasm. H represents Healthy, W represents Wounded

To further clarify the differential expression of defence- and sesquiterpene-related genes in ordinary *A. sinensis* and *Chi-Nan* germplasm under wounding stress, qRT–PCR analysis was performed. Overall, 15 DEGs were selected, including 4 candidate genes (*SCA15983.33*, *SCA128967.3*, *SCA104463.10* and *SCA135009.11.add*). As shown in Fig. 7, the expression patterns of both qRT-PCR and RNA-seq data were highly consistent. Similar to RNA-seq data, the gene expression of 4 candidate genes were all up-regulated in wounded *Chi-Nan* germplasm. These gene expression patterns were consistent with sesquiterpene biosynthesis content, which was possible to develop a new molecular marker for rapidly breeding *Chi-Nan* germplasm.

## Discussion

Agarwood is a traditional Chinese herbal medicine and precious spice known as “gold in medicine” and “king of incense”. As a the plant resource, *A. sinensis* can produce agarwood after injury [4]. *Chi-Nan* germplasm is a new *A. sinensis* germplasm with high agarwood-producing capacity [12]. The extension and planting of *Chi-Nan* germplasm can dramatically improve agarwood yield and quality. In this study, ordinary *A. sinensis* and *Chi-Nan* germplasm resources were simultaneously treated with cutting. After 30 d, micro-structure observation and sesquiterpenoid detection showed that more oil and sesquiterpene substances were produced in *Chi-Nan* germplasm than in ordinary *A. sinensis* (Fig. 1). These results were consistent with those seen in practice, suggesting that the *Chi-Nan* germplasm is a highly responsive *Aquilaria* line.

To investigate the regulatory mechanism of the high agarwood-producing capacity in *Chi-Nan* germplasm and breed excellent agarwood-producing strains, we performed comparative RNA sequencing to reveal the differential gene expression between ordinary *A. sinensis* and *Chi-Nan* germplasm. Similar to the mechanism responding to heat stress in heat-resistant and heat-sensitive jujube cultivars [34] and *Plasmodiophora brassicae* infection infection in resistant and susceptible rapeseed lines [35], there were dramatic differences in the gene expression levels between the two germplasm resources in their responses to wounding stress. The differential expression analysis of RNA-seq data in ordinary *A. sinensis* and *Chi-Nan* germplasm showed that the number of DEGs after wounding treatment in ordinary *A. sinensis* was obviously higher than in *Chi-Nan* germplasm, indicating that there were more biological processes involved in the response to wounding stress in ordinary *A. sinensis* (Fig. 3A). GO function analysis also showed that there were more DEGs-enriched terms in ordinary *A. sinensis* were more than in *Chi-Nan* germplasm. Among the enriched terms, only a few terms involved in amino acid metabolism and transport function were similar, and the majority of the terms differed significantly between the two germplasm resources (Fig. 4A). Moreover, we analysed the difference in KEGG enrichment between the two germplasm resources under wounding stress. Although the “starch and sucrose metabolism”, “α-linolenic acid metabolism” “plant hormone signal transduction” and “terpenoid backbone biosynthesis” pathways were enriched in both germplasm resources, the number of genes enriched in each pathway was different (Fig. 4B, Table S3). These results confirmed that regulatory mechanism underlying the response to wounding stress in *Chi-Nan* germplasm is different from that in ordinary *A. sinensis*.

Sesquiterpene exist widely in plants and is an important secondary metabolism, which can resist biotic and abiotic stimuli to enhance plant resistance [36, 37]. Sesquiterpene is one of main components of agarwood and can only be formed in stems, branches or roots when *A. sinensis* is injured [6]. Similar to other species, sesquiterpene are synthesized though MVA and MEP pathways in *A. sinensis* [19, 20]. Since sesquiterpenoid biosynthesis is different between ordinary *A. sinensis* and *Chi-Nan* germplasm resources (Fig. 1B), the DEGs enriched in “terpene backbone biosynthesis” and “terpenoids synthase” were in the spotlight. It was showed that the MVA and MEP pathway genes were differently induced to expression in ordinary *A. sinensis* and *Chi-Nan* germplasm (Fig. 5B), which indicated that the wound response sites and signal transduction pattern are different between two germplasm resources. Terpene synthase is the key catalytic enzyme for the formation of the sesquiterpenoid skeleton, and whose diversity often determines the structural diversity of sesquiterpenoids [38]. The terpene synthase genes played different roles in the expression characteristics in the ordinary *A. sinensis* and *Chi-Nan* germplasm during early stage of wounding stress (Fig. 5B), which may be the key genes for the subsequent differences in resin and sesquiterpene biosynthesis between the two germplasm resources, but this mechanism requires further investigation.

Previous studies have confirmed that terpene synthase genes are activated by MYC2 that is a basic helix-loop-helix transcription factor, such as N-methyltransferase (PMT) gene in tobacco [39], NbTPS1 in whitefly [40] and TPS21/11 in *Arabidopsis* inflorescence [41]. MYC2, as a master regulator in the JA signaling pathway, regulates diverse aspects of JA responses, including JA-mediated defence response and secondary metabolites biosynthesis [42–44]. Coincidentally, JA pathway including AsMYC2 plays an important role in regulating defence response and inducing expression of the sesquiterpene synthase in *A. sinensis* [18, 27, 28]. The genes related to JA pathway were identified in this study, which were up-regulated expression in both ordinary *A. sinensis* and *Chi-Nan* germplasm, but gene expression levels were different (Fig. 5B). It was indicated that the two germplasm resources had similar defensive responses, but the sensitivity to wound response is different. In addition, oxygen metabolism also is a crucial signal to defense to external stimuli in plant [45]. In *A. sinensis*, H_2_O_2_ that is an important component in oxygen metabolism can activate JA pathway to promote sesquiterpene biosynthesis under wound stress [29, 33]. 14 genes related to oxygen metabolism were identified and the gene expression pattern were analysis in comparative transcriptome. In accordance with JA pathway genes, oxygen metabolism-related genes were induced to express in both ordinary *A. sinensis* and *Chi-Nan* germplasm, but the expression intensity of genes was different in two germplasm resources. The results further confirmed that defensive responses of ordinary *A. sinensis* and *Chi-Nan* germplasm is similarity under wounding stress, but the sensitivity is different so that the two germplasm resources could invoke different biological processes in response to wounding stress.

Based on the important regulatory role of above pathways in defense response and sesquiterpene biosynthesis in *A. sinensis*. We identified 13 candidate gene that were correlated with rich diversity of sesquiterpene in *Chi-Nan* germplasm during early stage of wounding stress according to WGCNA analysis. These genes may provide valuable information for direction of excellent agarwood-producing germplasm breeding rapidly and prospect.

## Conclusions

The RNA-Seq analysis at the early stage of wounding stress in two *A. sinensis* germplasm resources indicated that *Chi-Nan* germplasm invoked different biological processes than ordinary *A. sinensis* germplasm, and the expression of defence signal (JA pathway and oxygen metabolism) and sesquiterpene biosynthesis pathway genes was significantly different between the two germplasm resources. A total of 13 candidate genes related to defence and sesquiterpene biosynthase were obtained by WGCNA, among which 4 candidate have been verified by qRT-PCR to be highly expressed in *Chi-Nan* germplasm. Therefore, this study not only provided a basis for further understanding the molecular mechanism on wounding stress in *A. sinensis* to improve the application of *Chi-Nan* germplasm but also identified valuable and useful genes involved in the high agarwood-producing capacity of *Chi-Nan* germplasm. These genes could be helpful for the genetic improvement of excellent agarwood-producing germplasm breeding and improving artificial agarwood production methods.

## Methods

### Plant materials and treatments

The 6-year-old ordinary *A. sinensis* germplasm and *Chi-Nan* germplasm resources were grown in a test plot at the Hainan Branch of the Institute of Medicinal Plant Development in Haikou city, Hainan Province, China (E 110^o^25’, N 20^o^01’) under day/night temperatures of 30 ± 2/25 ± 3 °*C. Chi-Nan* germplasm was identified as *Aquilaria sinensis (Lour.) Spreng* by DNA barcoding technology. The trees grew well and evenly. Two mature brands were selected from each tree. The diameter of the branches selected was 1.0 ± 0.2 cm. The branches were treated by cutting and longitudinal scratching on the surface as a wounding treatment. Wounded branches were enclosed in transparent bags. Two-centimetre-long samples without bark were collected from the apical end of each treated stem after 6 h [46] and 30 d. Healthy branches were used control samples and directly cut and collected. Samples were rapidly collected, immersed in liquid nitrogen and stored at − 80 °C for transcriptome sequencing and experiments. Three repetitions of each treatment were performed.

### Observation of microscopic structure

The 12 samples were cut into blocks (0.5 ± 0.1 mm^3^). The blocks were secured to the tray with frozen section embedding agent (Leica, Germany), which then placed in a Leica CM 1950 frozen microtome (Leica, Germany) to cool at a low temperature. Then, the secured blocks were sliced into slices of 50 μm along transverse. The slices was soaked in chloral hydrate transparent solution (Merck, USA) for a few minutes and laid flat on the slide. The structure of all slides was observed under a Nikon 80i light microscope (Nikon, Japan).

### Detection of volatile sesquiterpenoids in branches

Sesquiterpenoids from branches were extracted and detected according to research [29]. Frozen samples were ground to powder in liquid nitrogen with a Tissuelyser II grinding machine (Qiagen, Germany). The powder (0.5 g) was homogenized in diethyl ether (Xilong, China) and ultrasonicated on ice (Scientz, China), and the supernatant was collected. The supernatant was dried by a nitrogen blowing instrument (Hengyi, China) and dissolved in 1 ml diethyl ether (Xilong China). The extracts were filtered through a 0.45-μm membrane (Agilent Technologies, USA) for detection.

An Agilent 7890 A (Agilent, USA) equipped with an HP-5MS 5% Phenyl Silox capillary column (internal diameter, 30 m × 0.25 mm; film thickness, 0.25 μm) (Agilent, USA) was applied to test for volatile sesquiterpenoids in the extracts. The injection temperature was 240 °C, and the column temperature was initially held at 60 °C for 2 min and then increased to 250 °C at a rate of 4 °C min^− 1^. Helium was the carrier gas, whose flow rate was 1 mL/min. Identification of sesquiterpenoids was based on the NIST library with a matching degree greater than 60%. There were three independent repetitions of each biological experiment.

### RNA extraction and construction of cDNA library

The RNA extraction and construction of cDNA library were performed by Novogene company according to previous research [47]. In briefly, Total RNA was extracted from 12 samples using TRIzol reagent (Life Technologies, USA). RNA integrity was assessed using the RNA Nano 6000 Assay Kit for the Bioanalyzer 2100 system (Agilent Technologies, USA). One microgram of RNA per sample was used as the input material for the RNA sample preparations. mRNA was purified from total RNA using poly-T oligo-attached magnetic beads (Illumina, USA). Fragmentation was carried out using divalent cations under elevated temperature in First-Strand Synthesis Reaction Buffer (5×). First-strand cDNA was synthesized using random hexamer primers and M-MLV Reverse Transcriptase (RNase H-). Second-strand cDNA synthesis was subsequently performed using DNA Polymerase I and RNase H. Remaining overhangs were converted into blunt ends via exonuclease/polymerase activities. After adenylation of the 3′ ends of DNA fragments, adaptors with hairpin loop structures were ligated to prepare for hybridization. To preferentially select cDNA fragments 370 ~ 420 bp in length, the library fragments were purified with an AMPure XP system (Beckman Coulter, USA). Then, PCR was performed with Phusion High-Fidelity DNA polymerase, universal PCR primers and an index (X) primer. Finally, PCR products were purified by the AMPure XP system (Beckman Coulter, USA), and library quality was assessed on the Agilent Bioanalyzer 2100 system (Agilent Technologies, USA). Once the insert size met expectations, qRT-PCR was used to accurately quantify the effective concentration of the library (> 2 nM).

### RNA sequencing and data preprocessing

The Illumina HiSeq platform was used to sequence all 12 libraries, and 150 bp paired-end raw reads were generated. After filtering, high-quality clean data were obtained by removing reads containing adapters, reads containing poly-N sequences and low-quality reads from the raw data [48]. Then, the Q20, Q30 and GC contents of the clean data were calculated. High-quality clean data were considered for subsequent analyses. The high-quality paired-end clean reads were aligned to the *Aquilaria sinensis (Lour.) Spreng* reference genome (BioProject ID: PRJNA524272) using HISAT2 v2.0.5. Feature Counts v1.5.0-p3 was applied to count the read numbers mapped to each gene. Then, the fragment per kilobase of transcript per million reads (FPKM) of each gene was calculated based on the length of the gene and read count mapped to this gene. Microsoft Excel 2010 software was used to calculate the Pearson correlation of biological replicates. Principal component analysis (PCA) was performed with the linear algebra method, in which dimension reduction and principal component extraction of FPKM values for all genes were carried out.

### Differential expression and functional analysis

Differential expression analysis of different libraries was performed using the FPKM method, and the DESeq2R package (1.20.0) was used to identify the differentially expressed genes (DEGs) [49, 50]. Genes with an adjusted *P* value ≤0.05 and |log2 (fold change)| > 2 were assigned as DEGs [49]. Gene Ontology (GO) enrichment analysis of the DEGs was implemented by the ClusterProfiler R package (3.4.4), in which gene length bias was corrected. GO terms with a corrected P value ≤0.05 were considered significantly enriched by the DEGs. Kyoto Encyclopedia of Genes and Genomes (KEGG), which is a database resource for understanding high-level functions and utilities of the biological system, were carried out online (http://www.genome.jp/kegg/) [51]. The ClusterProfiler R package (3.4.4) was used to test the statistical enrichment of differentially expressed genes in the KEGG pathways. Genesis software was used to create heatmaps of the DEGs.

### Coexpression network analysis

Weighted correlation network analysis (WGCNA) is a systematic biological method used to describe the associations between genes among different samples [52]. The weighted association analysis used for network construction, gene screening, gene cluster identification, topological feature calculation, data simulation and visualization in WGCNA was conducted by the R package WGCNA (1.61). Module identification was implemented after merging modules in which expression profiles were similar.

### Quantitative real-time PCR (qRT-PCR) validation

The same samples of RNA were used for both RNA-seq and qRT–PCR verification. The RNA samples were reverse transcribed into cDNA using TransScript® One-Step gDNA Removal and cDNA Synthesis SuperMix (Transgene, China). The analysis was performed using TransStart Top Green qPCR SuperMix (Transgene, China) and a LightCycler®96 (Roche Switzerland). The transcript abundance was calculated from three biological and three technical replicates with *AsGAPDH* as an internal control [53]. The fold change was estimated using the 2^−ΔΔCT^ method [54]. The gene-specific primer sequences are listed in (Table S1).

## Supplementary Information

**Additional file 1: Table S1.** Primers used for quantitative RT-PCR. **Table S2.** Chemical composition and relative amounts of sesquiterpenoids from branches in two germplasm resources at 30 d after wounding stress. **Table S3.** Sequencing and mapping statistics for the 12 transcriptomes data. **Table S4.** Enriched KEGG pathways in two germplasm resources comparisons. **Table S5.** DEGs were enriched in KEGG pathways in green, turquoise and red modules.

## Data Availability

The raw transcriptome reads have been deposited into NCBI Short Read Archive (SRA) under accession number PRJNA819559.
