# Supplementary material for: Comparative transcriptome analysis reveals different defence responses during the early stage of wounding stress in Chi-Nan germplasm and ordinary Aquilaria sinensis
Source: BMC Plant Biol. 2022 Sep 29;22:464. doi: 10.1186/s12870-022-03821-4 (PMC9520901; doi:10.1186/s12870-022-03821-4)
Supplement: Supplementary file 1 — Additional file 1: Table S1. Primers used for quantitative RT-PCR. Table S2. Chemical composition and relative amounts of sesquiterpenoids from branches in two germplasm resources at 30 d after wounding stress. Table S3. Sequencing and mapping statistics for the 12 transcriptomes data. Table S4. Enriched KEGG pathways in two germplasm resources comparisons. Table S5. DEGs were enriched in KEGG pathways in green, turquoise and red modules. [file 12870_2022_3821_MOESM1_ESM.docx]

**Table S1 Primers used for quantitative RT-PCR**

| **No.** | **unigenes** | **Primers (5’ to 3’)** | |
| --- | --- | --- | --- |
| 1 | *SCA112107.94* | Forward | ACAGACCGTCAATGGAGTAAAG |
|  |  | Reverse | CCAGGGGACAAACCACTAAAA |
| 2 | *SCA42689.23* | Forward | TACCAAATGAACTGCCGATGC |
|  |  | Reverse | CACGCTTGAAGTAACCTGAAAAC |
| 3 | *SCA92025.20* | Forward | CATTTACGCCTGGTATCTGGACA |
|  |  | Reverse | TGATGACTTGAGGGATTTCATCG |
| 4 | *SCA7811.99* | Forward | GCTCGTGTCTATGTTCCTCCC |
|  |  | Reverse | CGCCGCCAAGTTCTTTATTCT |
| 5 | *SCA66331.3* | Forward | GTTGGGGACGAGTTGGGTG |
|  |  | Reverse | TGGATTGGTGGGGTTGGTT |
| 6 | *SCA117607.121* | Forward | GCTCCCCTGCTGACTATTGG |
|  |  | Reverse | ACGAGATTATGGCACGCCTC |
| 7 | *SCA15983.33* | Forward | GTGGAGGGGTCACAGTAGCA |
|  |  | Reverse | TTCTCGAACACCTTCGGATC |
| 8 | *SCA125647.74* | Forward | GAAGCGACGGCGTGGTAA |
|  |  | Reverse | GCAATGGCAGATTTGAGGC |
| 9 | *SCA33935.16* | Forward | CATACACCCAATACTTCGCTGAC |
|  |  | Reverse | ATGAGTAGATGGCGCAGAACG |
| 10 | *SCA50665.126* | Forward | CTATCATCCGCCCTTCTTCAG |
|  |  | Reverse | CTCCTCGTCTTCCTCCGTTGT |
| 11 | *SCA112107.71* | Forward | CGTAGTCGTTGCCGTTCCTC |
|  |  | Reverse | CTTCCTGCTTAACAAAGGTGGG |
| 12 | *SCA135009.11.add* | Forward | TGCTAAAGAAGAGGTGAAAAGGG |
|  |  | Reverse | CCGCAAGGTCGTCATAGAGC |
| 13 | *SCA27671.3* | Forward | AGGTTGTCCTCCCAGTTTCTGC |
|  |  | Reverse | GTATCCGCCGCAGCATCC |
| 14 | *SCA104463.10* | Forward | TGCTAGGGTTTCTATTGGGA |
|  |  | Reverse | CCATGGAGGTAGGTTGTCTG |
| 15 | *SCA128967.3* | Forward | GCATTTCGCTGCTGTTTC |
|  |  | Reverse | AATGGATTTGAGGTGGGTC |

**Table S2 Chemical composition and relative amounts of sesquiterpenoids from branches in two germplasm resources at 30 d after wounding stress**

| Peak | composition | *Chi-Nan germplasm* | | Ordinary*A.sinensis* | |
| --- | --- | --- | --- | --- | --- |
|  |  | retention time (min) | Peak areas  (×10^5^) | retention time (min) | Peak areas  (×10^5^) |
| 1 | α-Guaiene | 31.716 | 7.9±0.15^bcd^ |  |  |
| 2 | β-Humulene | 32.155 | 7.6±0.05^bcde^ | 32.184 | 6.55±0.15^cdef^ |
| 3 | β-Selinene | 32.386 | 5.95±0.225^ef^ |  |  |
| 4 | γ-eudesmol | 33.576 | 5.4±0.05^b^ | 33.565 | 5.2±0.3^ef^ |
| 5 | Caryophyllene oxide | 34.391 | 8.6±0.25^f^ |  |  |
| 6 | Aromandendrene |  |  | 37.655 | 6.05±0.25^f^ |
| 7 | α-Farnesene | 38.642 | 6.5±0.05^def^ | 38.631 | 6.55±0.45^cdef^ |
| 8 | Longifolenaldehyde | 39.717 | 8.3±0.2^bc^ | 39.711 | 6.4±0.2^def^ |
| 9 | Isoaromadendrene | 40.982 | 6.0±0.1^ef^ | 40.941 | 7.95±2.35^bcd^ |
| 10 | Caryophyllene-(I1) | 42.628 | 10.6±0^a^ |  |  |
| 11 | Alloaromadendrene | 45.401 | 7.25±1.325^bcde^ |  |  |

The different superscript letter are significantly different according to Duncan’s multiple range test (P<0.05)

**Table S3 Sequencing and mapping statistics for the 12 transcriptomes data**

| **Sample** | **Total raw reads** | **Total clean reads** | **Cleanbases** | **Q30**  **(%)** | **GC**  **(%)** | **Total**  **map** | **Uniquemap** |
| --- | --- | --- | --- | --- | --- | --- | --- |
| HKB_H_1 | 47115210 | 45768858 | 6.87G | 94.15 | 46.89 | 43266092  (94.53%) | 41304019  (90.24%) |
| HKB_H_2 | 46366552 | 45497050 | 6.82G | 94.01 | 47.87 | 43316380  (95.21%) | 39449567  (86.71%) |
| HKB_H_3 | 47081652 | 46271832 | 6.94G | 93.85 | 47.18 | 44200695  (95.52%) | 41979042  (90.72%) |
| HKB_W_1 | 47363864 | 46197440 | 6.93G | 93.66 | 47.83 | 43490589  (94.14%) | 39207341  (84.87%) |
| HKB_W_2 | 46879170 | 45953422 | 6.89G | 93.99 | 47.97 | 43925721  (95.59%) | 40571053  (88.29%) |
| HKB_W_3 | 46055300 | 44701084 | 6.71G | 94.29 | 47.8 | 42884469  (95.94%) | 40149697  (89.82%) |
| WZX_H_1 | 39994268 | 38522650 | 5.78G | 94.39 | 47.55 | 36198882  (93.97%) | 33616181  (87.26%) |
| WZX_H_2 | 34449228 | 32839802 | 4.93G | 94.98 | 48.2 | 30125157  (91.73%) | 27093494  (82.5%) |
| WZX_H_3 | 43485014 | 41853744 | 6.28G | 95.5 | 47.98 | 39191196  (93.64%) | 35366481  (84.5%) |
| WZX_W_1 | 44100210 | 41885706 | 6.28G | 95.44 | 48.77 | 39362290  (93.98%) | 35126849  (83.86%) |
| WZX_W_2 | 47223698 | 45297520 | 6.79G | 95.31 | 47.24 | 42796492  (94.48%) | 40335640  (89.05%) |
| WZX_W_3 | 42774294 | 40962880 | 6.14G | 94.42 | 48.05 | 38610986  (94.26%) | 34921101  (85.25%) |

**Table S4 Enriched KEGG pathways in two germplasm resources comparisons.**

| **No.** | **KEGGID** | **Description** | **Ordinary*A.sinensis*** | | ***Chi-Nan germplasm*** | |
| --- | --- | --- | --- | --- | --- | --- |
|  |  |  | **Up** | **down** | **up** | **down** |
| 1 | tcc03010 | Ribosome | 76 | 6 | 18 | 4 |
| 2 | tcc03008 | Ribosome biogenesis in eukaryotes | 29 | 2 | 6 | 0 |
| 3 | tcc00500 | Starch and sucrose metabolism | 21 | 22 | 14 | 12 |
| 4 | tcc00400 | Phenylalanine, tyrosine and tryptophan biosynthesis | 14 | 4 | 11 | 0 |
| 5 | tcc00860 | Porphyrin and chlorophyll metabolism | 7 | 13 | 1 | 1 |
| 6 | tcc04075 | Plant hormone signal transduction | 27 | 34 | 19 | 10 |
| 7 | tcc00960 | Tropane, piperidine and pyridine alkaloid biosynthesis | 6 | 5 | 6 | 0 |
| 8 | tcc00904 | Diterpenoid biosynthesis | 3 | 6 | 2 | 1 |
| 9 | tcc04016 | MAPK signaling pathway - plant | 30 | 8 | 12 | 1 |
| 10 | tcc00780 | Biotin metabolism | 7 | 1 | 3 | 1 |
| 11 | tcc00360 | Phenylalanine metabolism | 8 | 6 | 9 | 0 |
| 12 | tcc00350 | Tyrosine metabolism | 11 | 5 | 10 | 0 |
| 13 | tcc00950 | Isoquinoline alkaloid biosynthesis | 6 | 4 | 6 | 0 |
| 14 | tcc00240 | Pyrimidine metabolism | 12 | 7 | 1 | 1 |
| 15 | tcc00270 | Cysteine and methionine metabolism | 22 | 9 | 16 | 1 |
| 16 | tcc00590 | Arachidonic acid metabolism | 4 | 2 | 1 | 1 |
| 17 | tcc04626 | Plant-pathogen interaction | 34 | 7 | 14 | 5 |
| 18 | tcc00100 | Steroid biosynthesis | 4 | 8 | 5 | 0 |
| 19 | tcc00020 | Citrate cycle (TCA cycle) | 13 | 2 | 8 | 2 |
| 20 | tcc00940 | Phenylpropanoid biosynthesis | 24 | 10 | 19 | 5 |
| 21 | tcc01230 | Biosynthesis of amino acids | 49 | 10 | 33 | 7 |
| 22 | tcc00906 | Carotenoid biosynthesis | 2 | 10 | 0 | 2 |
| 23 | tcc00195 | Photosynthesis | 3 | 12 | 1 | 0 |
| 24 | tcc01212 | Fatty acid metabolism | 17 | 4 | 9 | 2 |
| 25 | tcc00592 | alpha-Linolenic acid metabolism | 12 | 2 | 6 | 1 |
| 26 | tcc01210 | 2-Oxocarboxylic acid metabolism | 11 | 6 | 7 | 3 |
| 27 | tcc04712 | Circadian rhythm - plant | 6 | 5 | 0 | 1 |
| 28 | tcc00330 | Arginine and proline metabolism | 10 | 8 | 9 | 3 |
| 29 | tcc00061 | Fatty acid biosynthesis | 10 | 2 | 5 | 2 |
| 30 | tcc00908 | Zeatin biosynthesis | 3 | 3 | 3 | 1 |
| 31 | tcc01040 | Biosynthesis of unsaturated fatty acids | 5 | 1 | 3 | 0 |
| 32 | tcc00480 | Glutathione metabolism | 13 | 8 | 6 | 6 |
| 33 | tcc01200 | Carbon metabolism | 41 | 19 | 22 | 6 |
| 34 | tcc00760 | Nicotinate and nicotinamide metabolism | 4 | 3 | 0 | 2 |
| 35 | tcc00670 | One carbon pool by folate | 5 | 1 | 2 | 1 |
| 36 | tcc00511 | Other glycan degradation | 2 | 5 | 3 | 0 |
| 37 | tcc00790 | Folate biosynthesis | 4 | 3 | 1 | 1 |
| 38 | tcc00052 | Galactose metabolism | 9 | 3 | 6 | 0 |
| 39 | tcc00250 | Alanine, aspartate and glutamate metabolism | 6 | 7 | 4 | 1 |
| 40 | tcc00941 | Flavonoid biosynthesis | 7 | 1 | 7 | 0 |
| 41 | tcc02010 | ABC transporters | 2 | 4 | 1 | 2 |
| 42 | tcc00620 | Pyruvate metabolism | 11 | 10 | 8 | 1 |
| 43 | tcc00730 | Thiamine metabolism | 4 | 1 | 5 | 2 |
| 44 | tcc00565 | Ether lipid metabolism | 5 | 3 | 6 | 2 |
| 45 | tcc00640 | Propanoate metabolism | 9 | 2 | 5 | 1 |
| 46 | tcc00071 | Fatty acid degradation | 11 | 3 | 8 | 1 |
| 47 | tcc00750 | Vitamin B6 metabolism | 3 | 1 | 2 | 2 |
| 48 | tcc00260 | Glycine, serine and threonine metabolism | 12 | 4 | 8 | 1 |
| 49 | tcc00920 | Sulfur metabolism | 6 | 2 | 6 | 2 |
| 50 | tcc00196 | Photosynthesis - antenna proteins | 0 | 5 | 1 | 1 |
| 51 | tcc00290 | Valine, leucine and isoleucine biosynthesis | 4 | 1 | 1 | 0 |
| 52 | tcc00450 | Selenocompound metabolism | 4 | 0 | 3 | 1 |
| 53 | tcc00531 | Glycosaminoglycan degradation | 1 | 3 | 0 | 2 |
| 54 | tcc00710 | Carbon fixation in photosynthetic organisms | 7 | 7 | 3 | 1 |
| 55 | tcc00630 | Glyoxylate and dicarboxylate metabolism | 8 | 7 | 5 | 2 |
| 56 | tcc00040 | Pentose and glucuronateinterconversions | 8 | 8 | 9 | 1 |
| 57 | tcc00230 | Purine metabolism | 17 | 4 | 8 | 4 |
| 58 | tcc04146 | Peroxisome | 16 | 4 | 7 | 3 |
| 59 | tcc00051 | Fructose and mannose metabolism | 8 | 4 | 6 | 3 |
| 60 | tcc00380 | Tryptophan metabolism | 6 | 4 | 6 | 1 |
| 61 | tcc00460 | Cyanoamino acid metabolism | 6 | 1 | 5 | 1 |
| 62 | tcc00910 | Nitrogen metabolism | 4 | 3 | 1 | 4 |
| 63 | tcc00520 | Amino sugar and nucleotide sugar metabolism | 18 | 7 | 22 | 2 |
| 64 | tcc03440 | Homologous recombination | 7 | 7 | 1 | 6 |
| 65 | tcc00130 | Ubiquinone and other terpenoid-quinone biosynthesis | 7 | 3 | 6 | 2 |
| 66 | tcc03020 | RNA polymerase | 6 | 3 | 2 | 1 |
| 67 | tcc00220 | Arginine biosynthesis | 3 | 5 | 4 | 2 |
| 68 | tcc00340 | Histidine metabolism | 4 | 1 | 5 | 2 |
| 69 | tcc00561 | Glycerolipid metabolism | 9 | 9 | 13 | 4 |
| 70 | tcc00740 | Riboflavin metabolism | 1 | 3 | 1 | 1 |
| 71 | tcc00010 | Glycolysis / Gluconeogenesis | 18 | 8 | 14 | 3 |
| 72 | tcc00300 | Lysine biosynthesis | 3 | 0 | 2 | 1 |
| 73 | tcc00410 | beta-Alanine metabolism | 7 | 4 | 7 | 2 |
| 74 | tcc00030 | Pentose phosphate pathway | 8 | 2 | 4 | 0 |
| 75 | tcc00600 | Sphingolipid metabolism | 2 | 4 | 2 | 0 |
| 76 | tcc00062 | Fatty acid elongation | 3 | 2 | 4 | 1 |
| 77 | tcc00280 | Valine, leucine and isoleucine degradation | 8 | 4 | 11 | 2 |
| 78 | tcc00900 | Terpenoid backbone biosynthesis | 7 | 3 | 7 | 2 |
| 79 | tcc03060 | Protein export | 8 | 2 | 4 | 1 |
| 80 | tcc03030 | DNA replication | 7 | 3 | 3 | 3 |
| 81 | tcc00513 | Various types of N-glycan biosynthesis | 6 | 1 | 4 | 0 |
| 82 | tcc00650 | Butanoate metabolism | 4 | 0 | 3 | 0 |
| 83 | tcc00770 | Pantothenate and CoA biosynthesis | 4 | 4 | 5 | 2 |
| 84 | tcc00073 | Cutin, suberine and wax biosynthesis | 4 | 0 | 0 | 1 |
| 85 | tcc03430 | Mismatch repair | 5 | 2 | 2 | 4 |
| 86 | tcc04141 | Protein processing in endoplasmic reticulum | 28 | 6 | 21 | 3 |
| 87 | tcc04145 | Phagosome | 6 | 7 | 7 | 0 |
| 88 | tcc00514 | Other types of O-glycan biosynthesis | 1 | 1 | 1 | 0 |
| 89 | tcc03420 | Nucleotide excision repair | 8 | 2 | 3 | 4 |
| 90 | tcc04070 | Phosphatidylinositol signaling system | 5 | 5 | 2 | 3 |
| 91 | tcc00510 | N-Glycan biosynthesis | 8 | 0 | 4 | 1 |
| 92 | tcc03022 | Basal transcription factors | 6 | 2 | 0 | 4 |
| 93 | tcc03050 | Proteasome | 7 | 1 | 4 | 3 |
| 94 | tcc03018 | RNA degradation | 19 | 1 | 5 | 5 |
| 95 | tcc00053 | Ascorbate and aldarate metabolism | 3 | 4 | 12 | 5 |
| 96 | tcc00564 | Glycerophospholipid metabolism | 11 | 6 | 12 | 7 |
| 97 | tcc00562 | Inositol phosphate metabolism | 5 | 6 | 5 | 3 |
| 98 | tcc04120 | Ubiquitin mediated proteolysis | 10 | 8 | 1 | 0 |
| 99 | tcc03013 | RNA transport | 26 | 2 | 2 | 8 |
| 100 | tcc03410 | Base excision repair | 3 | 1 | 4 | 1 |
| 101 | tcc00310 | Lysine degradation | 3 | 1 | 6 | 1 |
| 102 | tcc04136 | Autophagy - other | 5 | 0 | 1 | 1 |
| 103 | tcc04130 | SNARE interactions in vesicular transport | 4 | 1 | 1 | 1 |
| 104 | tcc00190 | Oxidative phosphorylation | 10 | 16 | 9 | 1 |
| 105 | tcc03040 | Spliceosome | 22 | 7 | 2 | 18 |
| 106 | tcc04144 | Endocytosis | 12 | 11 | 13 | 5 |
| 107 | tcc00970 | Aminoacyl-tRNA biosynthesis | 5 | 4 | 1 | 5 |
| 108 | tcc03015 | mRNA surveillance pathway | 9 | 4 | 2 | 11 |
| 109 | tcc00563 | Glycosylphosphatidylinositol (GPI)-anchor biosynthesis | 1 | 0 |  |  |
| 110 | tcc00905 | Brassinosteroid biosynthesis | 4 | 2 |  |  |

**Table S5 DEGs were enriched in KEGG pathways in green, turquoise and red modules.**

| KEGGID | Description | geneID | module |
| --- | --- | --- | --- |
| tcc00561 | Glycerolipid metabolism | SCA142385.116/SCA77405.54/SCA91791.3/SCA85249.57/SCA74405.23/SCA100847.48/SCA12551.7 | green |
| tcc00280 | Valine, leucine and isoleucine degradation | SCA77405.54/SCA140505.31/SCA115273.12/SCA91791.3/SCA118161.19 | green |
| tcc00564 | Glycerophospholipid metabolism | SCA142385.116/SCA132811.46/SCA74405.23/SCA100847.48/SCA113111.4/SCA12551.7 | green |
| tcc00062 | Fatty acid elongation | SCA142139.27/SCA46613.211/SCA116795.68 | green |
| tcc00941 | Flavonoid biosynthesis | SCA73683.9/SCA137375.1/SCA39707.21 | green |
| tcc00770 | Pantothenate and CoA biosynthesis | SCA77405.54/SCA140505.31/SCA91791.3 | green |
| tcc00053 | Ascorbate and aldarate metabolism | SCA77405.54/SCA91791.3/SCA103829.54 | green |
| tcc00900 | Terpenoid backbone biosynthesis | SCA113871.37/SCA50665.128/SCA18745.55 | green |
| tcc00340 | Histidine metabolism | SCA77405.54/SCA91791.3 | green |
| tcc04626 | Plant-pathogen interaction | SCA142139.27/SCA132985.1/SCA6397.56/SCA46613.211/SCA116795.68 | green |
| tcc00310 | Lysine degradation | SCA77405.54/SCA91791.3 | green |
| tcc00100 | Steroid biosynthesis | SCA112107.120/SCA86445.4 | green |
| tcc00640 | Propanoate metabolism | SCA115273.12/SCA118161.19 | green |
| tcc00410 | beta-Alanine metabolism | SCA77405.54/SCA91791.3 | green |
| tcc00592 | alpha-Linolenic acid metabolism | SCA15983.33/SCA136357.10 | green |
| tcc04075 | Plant hormone signal transduction | SCA47097.151/SCA122967.15/SCA131617.54/SCA137391.19/SCA614316.1 | green |
| tcc00380 | Tryptophan metabolism | SCA77405.54/SCA91791.3 | green |
| tcc00071 | Fatty acid degradation | SCA77405.54/SCA91791.3 | green |
| tcc00330 | Arginine and proline metabolism | SCA77405.54/SCA91791.3 | green |
| tcc04141 | Protein processing in endoplasmic reticulum | SCA83573.101/SCA132985.1/SCA69821.92/SCA112873.56 | green |
| tcc00010 | Glycolysis / Gluconeogenesis | SCA77405.54/SCA91791.3/SCA85249.57 | green |
| tcc00904 | Diterpenoid biosynthesis | SCA82909.58 | green |
| tcc00290 | Valine, leucine and isoleucine biosynthesis | SCA140505.31 | green |
| tcc00908 | Zeatin biosynthesis | SCA117607.30 | green |
| tcc02010 | ABC transporters | SCA139903.33 | green |
| tcc00500 | Starch and sucrose metabolism | SCA131089.105/SCA115307.43/SCA34817.2 | green |
| tcc00730 | Thiamine metabolism | SCA112193.146 | green |
| tcc00940 | Phenylpropanoid biosynthesis | SCA12335.3/SCA142457.1/SCA46613.112 | green |
| tcc00620 | Pyruvate metabolism | SCA77405.54/SCA91791.3 | green |
| tcc00230 | Purine metabolism | SCA112193.146/SCA63757.137 | green |
| tcc00565 | Ether lipid metabolism | SCA132811.46 | green |
| tcc00040 | Pentose and glucuronate interconversions | SCA33713.2/SCA85249.57 | green |
| tcc00520 | Amino sugar and nucleotide sugar metabolism | SCA132155.27/SCA74405.12 | green |
| tcc00350 | Tyrosine metabolism | SCA140733.1 | green |
| tcc00250 | Alanine, aspartate and glutamate metabolism | SCA91487.43 | green |
| tcc00052 | Galactose metabolism | SCA44767.12 | green |
| tcc01210 | 2-Oxocarboxylic acid metabolism | SCA140505.31 | green |
| tcc00562 | Inositol phosphate metabolism | SCA103829.54 | green |
| tcc04144 | Endocytosis | SCA132811.46/SCA75437.74 | green |
| tcc00270 | Cysteine and methionine metabolism | SCA140505.31 | green |
| tcc01230 | Biosynthesis of amino acids | SCA140505.31/SCA91487.43 | green |
| tcc04016 | MAPK signaling pathway - plant | SCA614488.1/SCA40961.2/SCA50665.96/SCA107503.18/SCA126049.115/SCA137375.60/SCA142385.149/SCA614854.1/SCA97369.21/SCA32123.13/SCA132155.1/SCA53313.45/SCA47169.9/SCA129853.5/SCA30763.125/SCA90769.20/SCA109309.20 | turquoise |
| tcc00940 | Phenylpropanoid biosynthesis | SCA58341.10/SCA136793.31/SCA7811.183/SCA26857.32/SCA17141.101/SCA133811.97/SCA56723.9/SCA24387.3/SCA65009.91/SCA116197.1/SCA133811.157/SCA142457.14/SCA45021.14/SCA47911.9/SCA91071.16/SCA133811.144 | turquoise |
| tcc04626 | Plant-pathogen interaction | SCA614488.1/SCA40961.2/SCA50665.96/SCA126049.115/SCA142385.149/SCA97369.21/SCA128681.118/SCA32123.13/SCA17859.25/SCA108547.11/SCA132155.1/SCA29571.30/SCA47169.9/SCA129853.5/SCA109309.20/SCA118667.16 | turquoise |
| tcc00500 | Starch and sucrose metabolism | SCA136793.31/SCA70199.20/SCA129495.6/SCA121315.8/SCA133811.195/SCA90769.100/SCA46613.154/SCA71553.34/SCA140017.17/SCA138051.65/SCA9557.72/SCA7799.43 | turquoise |
| tcc00330 | Arginine and proline metabolism | SCA66331.8/SCA69821.77/SCA61963.16/SCA104821.63/SCA120787.6/SCA129495.12/SCA91465.3 | turquoise |
| tcc00592 | alpha-Linolenic acid metabolism | SCA7811.12/SCA66331.31/SCA117607.52/SCA86359.4/SCA92025.19/SCA117607.121 | turquoise |
| tcc00270 | Cysteine and methionine metabolism | SCA107503.18/SCA132681.25/SCA61963.16/SCA104821.63/SCA88003.77/SCA6397.82/SCA118161.1/SCA76823.31/SCA135359.28/SCA48195.51 | turquoise |
| tcc00950 | Isoquinoline alkaloid biosynthesis | SCA61963.16/SCA88003.77/SCA138043.28/SCA134973.26 | turquoise |
| tcc00360 | Phenylalanine metabolism | SCA61963.16/SCA88003.77/SCA91465.3/SCA138043.28/SCA91071.16 | turquoise |
| tcc00905 | Brassinosteroid biosynthesis | SCA44767.72/SCA23199.31/SCA46007.36 | turquoise |
| tcc00195 | Photosynthesis | SCA109265.45/SCA19097.39/SCA69821.39/SCA140453.59/SCA74405.55/SCA614172.4 | turquoise |
| tcc00400 | Phenylalanine, tyrosine and tryptophan biosynthesis | SCA61963.16/SCA88003.77/SCA40753.1/SCA117981.1/SCA116789.4 | turquoise |
| tcc00920 | Sulfur metabolism | SCA140453.102_SCA140453.103/SCA6397.82/SCA612974.1/SCA48195.51 | turquoise |
| tcc00904 | Diterpenoid biosynthesis | SCA137187.5/SCA109691.2/SCA135359.9 | turquoise |
| tcc00908 | Zeatin biosynthesis | SCA140855.61/SCA29571.52/SCA127697.1 | turquoise |
| tcc00380 | Tryptophan metabolism | SCA16155.67/SCA69821.77/SCA23199.7/SCA91465.3/SCA138043.28 | turquoise |
| tcc00051 | Fructose and mannose metabolism | SCA117607.58/SCA33935.42/SCA71553.34/SCA140017.17/SCA138051.65 | turquoise |
| tcc00960 | Tropane, piperidine and pyridine alkaloid biosynthesis | SCA61963.16/SCA88003.77/SCA131715.107 | turquoise |
| tcc04075 | Plant hormone signal transduction | SCA139903.22/SCA50665.96/SCA136071.14/SCA107503.50/SCA34921.12/SCA83675.1/SCA614854.1/SCA56815.23/SCA14519.33/SCA34921.19/SCA136519.39/SCA30763.125/SCA90769.20 | turquoise |
| tcc00130 | Ubiquinone and other terpenoid-quinone biosynthesis | SCA26857.32/SCA88003.77/SCA116197.1/SCA142457.14 | turquoise |
| tcc00350 | Tyrosine metabolism | SCA61963.16/SCA88003.77/SCA138043.28/SCA134973.26 | turquoise |
| tcc00910 | Nitrogen metabolism | SCA97369.35/SCA112193.30/SCA55005.61 | turquoise |
| tcc00565 | Ether lipid metabolism | SCA112107.77/SCA33713.1/SCA58869.64 | turquoise |
| tcc00450 | Selenocompound metabolism | SCA6397.82/SCA51043.14 | turquoise |
| tcc01230 | Biosynthesis of amino acids | SCA33935.42/SCA132681.25/SCA61963.16/SCA99627.10/SCA6397.82/SCA128681.110/SCA140453.35/SCA40753.1/SCA117981.1/SCA95379.5/SCA135359.28/SCA48195.51/SCA116789.4 | turquoise |
| tcc00030 | Pentose phosphate pathway | SCA33935.42/SCA14901.10/SCA128681.110/SCA95379.5 | turquoise |
| tcc00052 | Galactose metabolism | SCA33935.42/SCA47555.103/SCA140017.17/SCA56487.1 | turquoise |
| tcc00071 | Fatty acid degradation | SCA7811.12/SCA69821.77/SCA23199.7/SCA86359.4 | turquoise |
| tcc00196 | Photosynthesis - antenna proteins | SCA20213.2/SCA56653.48 | turquoise |
| tcc00945 | Stilbenoid, diarylheptanoid and gingerol biosynthesis | SCA133811.157/SCA142457.14 | turquoise |
| tcc00941 | Flavonoid biosynthesis | SCA116795.84/SCA133811.157/SCA142457.14 | turquoise |
| tcc00280 | Valine, leucine and isoleucine degradation | SCA7811.12/SCA69821.77/SCA23199.7/SCA112107.71 | turquoise |
| tcc03440 | Homologous recombination | SCA65465.44/SCA83573.18/SCA18745.44/SCA29571.14 | turquoise |
| tcc00650 | Butanoate metabolism | SCA23199.7/SCA112107.71 | turquoise |
| tcc00620 | Pyruvate metabolism | SCA69821.77/SCA11531.9/SCA23199.7/SCA137375.48/SCA114761.12 | turquoise |
| tcc00790 | Folate biosynthesis | SCA116795.157/SCA97369.29 | turquoise |
| tcc00710 | Carbon fixation in photosynthetic organisms | SCA11531.9/SCA61963.16/SCA128681.110/SCA114761.12 | turquoise |
| tcc01200 | Carbon metabolism | SCA11531.9/SCA33935.42/SCA23199.7/SCA61963.16/SCA99627.10/SCA14901.10/SCA97369.35/SCA128681.110/SCA114761.12/SCA95379.5/SCA140017.17/SCA48195.51 | turquoise |
| tcc00520 | Amino sugar and nucleotide sugar metabolism | SCA142385.9/SCA613876.1/SCA71553.34/SCA45021.59/SCA140017.17/SCA138051.65 | turquoise |
| tcc00250 | Alanine, aspartate and glutamate metabolism | SCA142385.9/SCA61963.16/SCA97369.35 | turquoise |
| tcc00053 | Ascorbate and aldarate metabolism | SCA69821.77/SCA142139.23/SCA129195.8 | turquoise |
| tcc01212 | Fatty acid metabolism | SCA7811.12/SCA128681.57/SCA23199.7/SCA86359.4 | turquoise |
| tcc00073 | Cutin, suberine and wax biosynthesis | SCA142477.49/SCA18745.66 | turquoise |
| tcc00310 | Lysine degradation | SCA69821.77/SCA23199.7 | turquoise |
| tcc00220 | Arginine biosynthesis | SCA61963.16/SCA97369.35 | turquoise |
| tcc00100 | Steroid biosynthesis | SCA7811.146/SCA3333.1 | turquoise |
| tcc04712 | Circadian rhythm - plant | SCA9557.29/SCA14433.29 | turquoise |
| tcc01210 | 2-Oxocarboxylic acid metabolism | SCA16155.67/SCA132681.25/SCA61963.16 | turquoise |
| tcc00300 | Lysine biosynthesis | SCA132681.25 | turquoise |
| tcc03430 | Mismatch repair | SCA83573.18/SCA18745.44 | turquoise |
| tcc00260 | Glycine, serine and threonine metabolism | SCA132681.25/SCA99627.10/SCA140453.35 | turquoise |
| tcc00230 | Purine metabolism | SCA83573.122/SCA140453.102_SCA140453.103/SCA112107.8/SCA95379.5 | turquoise |
| tcc00780 | Biotin metabolism | SCA128681.57 | turquoise |
| tcc00040 | Pentose and glucuronate interconversions | SCA140947.1/SCA60525.8/SCA7677.16/SCA95297.18 | turquoise |
| tcc00010 | Glycolysis / Gluconeogenesis | SCA69821.77/SCA33935.42/SCA47555.103/SCA114761.12/SCA140017.17 | turquoise |
| tcc00909 | Sesquiterpenoid and triterpenoid biosynthesis | SCA93491.1 | turquoise |
| tcc03410 | Base excision repair | SCA83573.18/SCA97943.29 | turquoise |
| tcc00290 | Valine, leucine and isoleucine biosynthesis | SCA99627.10 | turquoise |
| tcc00410 | beta-Alanine metabolism | SCA69821.77/SCA120787.6 | turquoise |
| tcc00340 | Histidine metabolism | SCA69821.77 | turquoise |
| tcc00760 | Nicotinate and nicotinamide metabolism | SCA141753.17 | turquoise |
| tcc02010 | ABC transporters | SCA110113.5 | turquoise |
| tcc03030 | DNA replication | SCA83573.18/SCA18745.44 | turquoise |
| tcc00480 | Glutathione metabolism | SCA104821.63/SCA79533.11/SCA100847.21 | turquoise |
| tcc03008 | Ribosome biogenesis in eukaryotes | SCA7811.24/SCA613572.1/SCA54077.16 | turquoise |
| tcc00900 | Terpenoid backbone biosynthesis | SCA23199.7/SCA112107.71 | turquoise |
| tcc03420 | Nucleotide excision repair | SCA83573.18/SCA18745.44 | turquoise |
| tcc00062 | Fatty acid elongation | SCA29571.30 | turquoise |
| tcc00600 | Sphingolipid metabolism | SCA107077.37 | turquoise |
| tcc04146 | Peroxisome | SCA7811.12/SCA39707.40/SCA137375.56 | turquoise |
| tcc00240 | Pyrimidine metabolism | SCA116489.35/SCA142477.133 | turquoise |
| tcc00460 | Cyanoamino acid metabolism | SCA136793.31 | turquoise |
| tcc00906 | Carotenoid biosynthesis | SCA47097.51 | turquoise |
| tcc00564 | Glycerophospholipid metabolism | SCA112107.77/SCA33713.1/SCA58869.64 | turquoise |
| tcc00640 | Propanoate metabolism | SCA23199.7 | turquoise |
| tcc03020 | RNA polymerase | SCA7677.67 | turquoise |
| tcc00061 | Fatty acid biosynthesis | SCA128681.57 | turquoise |
| tcc04130 | SNARE interactions in vesicular transport | SCA134561.2 | turquoise |
| tcc03050 | Proteasome | SCA613456.1 | turquoise |
| tcc00860 | Porphyrin and chlorophyll metabolism | SCA17141.132 | turquoise |
| tcc04070 | Phosphatidylinositol signaling system | SCA126049.115 | turquoise |
| tcc03018 | RNA degradation | SCA33935.42/SCA12813.47 | turquoise |
| tcc00630 | Glyoxylate and dicarboxylate metabolism | SCA23199.7 | turquoise |
| tcc04120 | Ubiquitin mediated proteolysis | SCA8389.10/SCA106403.33 | turquoise |
| tcc04145 | Phagosome | SCA139607.23 | turquoise |
| tcc00190 | Oxidative phosphorylation | SCA139607.23/SCA35289.20 | turquoise |
| tcc04144 | Endocytosis | SCA46613.185/SCA112107.77/SCA33713.1 | turquoise |
| tcc00561 | Glycerolipid metabolism | SCA69821.77 | turquoise |
| tcc03010 | Ribosome | SCA7811.199/SCA142139.19/SCA100847.79/SCA73683.3/SCA17859.15 | turquoise |
| tcc03015 | mRNA surveillance pathway | SCA100847.7 | turquoise |
| tcc03013 | RNA transport | SCA100847.7 | turquoise |
| tcc03040 | Spliceosome | SCA100847.7 | turquoise |
| tcc04141 | Protein processing in endoplasmic reticulum | SCA18745.21 | turquoise |
| tcc00073 | Cutin, suberine and wax biosynthesis | SCA83675.2/SCA120025.1/SCA94133.37 | red |
| tcc00950 | Isoquinoline alkaloid biosynthesis | SCA70671.9/SCA15491.7 | red |
| tcc00941 | Flavonoid biosynthesis | SCA112107.88/SCA117607.62 | red |
| tcc00350 | Tyrosine metabolism | SCA70671.9/SCA15491.7 | red |
| tcc00190 | Oxidative phosphorylation | SCA98933.6/SCA104523.39/SCA125647.56 | red |
| tcc04145 | Phagosome | SCA73267.14/SCA140833.77 | red |
| tcc00905 | Brassinosteroid biosynthesis | SCA46613.68 | red |
| tcc00740 | Riboflavin metabolism | SCA136173.16 | red |
| tcc00040 | Pentose and glucuronate interconversions | SCA29571.16/SCA142385.2 | red |
| tcc00960 | Tropane, piperidine and pyridine alkaloid biosynthesis | SCA15491.7 | red |
| tcc04016 | MAPK signaling pathway - plant | SCA33015.13/SCA127385.6 | red |
| tcc00360 | Phenylalanine metabolism | SCA15491.7 | red |
| tcc00940 | Phenylpropanoid biosynthesis | SCA91487.144/SCA50665.86 | red |
| tcc00130 | Ubiquinone and other terpenoid-quinone biosynthesis | SCA91487.144 | red |
| tcc03022 | Basal transcription factors | SCA611728.1 | red |
| tcc03050 | Proteasome | SCA39065.44 | red |
| tcc03060 | Protein export | SCA73267.14 | red |
| tcc00410 | beta-Alanine metabolism | SCA15491.7 | red |
| tcc00592 | alpha-Linolenic acid metabolism | SCA135153.1 | red |
| tcc00380 | Tryptophan metabolism | SCA98011.19 | red |
| tcc04626 | Plant-pathogen interaction | SCA11729.25/SCA33015.13 | red |
| tcc00970 | Aminoacyl-tRNA biosynthesis | SCA53313.72 | red |
| tcc01210 | 2-Oxocarboxylic acid metabolism | SCA98011.19 | red |
| tcc00260 | Glycine, serine and threonine metabolism | SCA15491.7 | red |
| tcc03008 | Ribosome biogenesis in eukaryotes | SCA33713.30 | red |
| tcc04144 | Endocytosis | SCA140833.77 | red |
| tcc04141 | Protein processing in endoplasmic reticulum | SCA73267.14 | red |
